# The Dynamic Interface Between the Bone Marrow Vascular Niche and Hematopoietic Stem Cells in Myeloid Malignancy

**DOI:** 10.3389/fcell.2021.635189

**Published:** 2021-03-11

**Authors:** Laura Mosteo, Joanna Storer, Kiran Batta, Emma J. Searle, Delfim Duarte, Daniel H. Wiseman

**Affiliations:** ^1^Instituto de Investigação e Inovação em Saúde (i3S), University of Porto, Porto, Portugal; ^2^Epigenetics of Haematopoiesis Group, Division of Cancer Sciences, The University of Manchester, Manchester, United Kingdom; ^3^Department of Haematology, The Christie NHS Foundation Trust, Manchester, United Kingdom; ^4^Department of Biomedicine, Faculdade de Medicina da Universidade do Porto (FMUP), Porto, Portugal; ^5^Department of Onco-Hematology, Instituto Português de Oncologia (IPO)-Porto, Porto, Portugal

**Keywords:** vascular niche, hematopoietic stem cells, acute myeloid leukemia, myelodysplastic syndromes, targeted therapies

## Abstract

Hematopoietic stem cells interact with bone marrow niches, including highly specialized blood vessels. Recent studies have revealed the phenotypic and functional heterogeneity of bone marrow endothelial cells. This has facilitated the analysis of the vascular microenvironment in steady state and malignant hematopoiesis. In this review, we provide an overview of the bone marrow microenvironment, focusing on refined analyses of the marrow vascular compartment performed in mouse studies. We also discuss the emerging role of the vascular niche in “inflamm-aging” and clonal hematopoiesis, and how the endothelial microenvironment influences, supports and interacts with hematopoietic cells in acute myeloid leukemia and myelodysplastic syndromes, as exemplar states of malignant myelopoiesis. Finally, we provide an overview of strategies for modulating these bidirectional interactions to therapeutic effect in myeloid malignancies.

## Introduction

Throughout an organism’s lifespan somatic stem cells face the substantial challenge of maintaining highly regenerative tissues, such as blood, that are characterized by rapid and continuous cell turnover. In a healthy adult human, it is estimated that ∼1.5 million highly specialized blood cells are produced each second to fulfill diverse vital functions, including oxygen transport, defense against pathogens and initiation of coagulation. Mature blood cells are generated from hematopoietic stem cells (HSCs) in the complex and highly regulated process of hematopoiesis, which after early embryogenesis occurs predominantly within the bone marrow (BM). Self-renewal, proliferation, migration and differentiation of HSCs into different lineages are tightly regulated to maintain homeostasis in the steady state and under stress conditions. These coordinated cell fate decisions involve cell-intrinsic mechanisms (transcriptional, epigenetic, and metabolic), but also cell-extrinsic cues from the supportive BM microenvironment (Zon, 2008).

The HSC niche is defined as the cellular and molecular microenvironment wherein HSCs reside and from which they receive fundamental regulatory signals. Over recent decades many components of the niche, their secreted factors and associated functions and interactions have been identified. Mechanistic insights have been gleaned from murine studies, but specific roles in the regulation of HSC behavior and function in humans remain incompletely understood or controversial.

The BM represents the most studied and best characterized stem cell niche. Such work has largely relied on murine models, including several reporter mice and murine Cre lines generated to visualize and/or target different components of the BM microenvironment (summarized in Supplementary Table 1). However, these animal studies come with their inherent limitations, and study of the BM niche has been further hampered by a lack of specific and universal markers to reliably identify HSCs immunophenotypically, or to robustly distinguish, subclassify and enrich all relevant stromal elements. Until recently technical limitations were a major hindrance. For example, imaging platforms were restricted to few channels, with experiments typically performed under transplantation conditions involving pre-irradiation of recipient animals, thereby inducing profound alteration of the BM microenvironment and confounding interpretation of results. In recent years, however, imaging and flow cytometry technologies have advanced rapidly; in parallel, several reporter mice with increasing specificity for HSCs have been developed, allowing imaging of HSCs in their natural microenvironment and helping to resolve the controversy of *in vivo* HSC localization within the BM (Acar et al., 2015; Chen et al., 2016; Christodoulou et al., 2020; Upadhaya et al., 2020).

The HSC niche is frequently impaired in hematological malignancies and upon injury (e.g., by chemotherapy or radiation therapy), with resultant defective hematopoiesis and likely contributing to disease progression. Alterations in the BM niche also carry the potential to initiate disease in experimental models (Walkley et al., 2007; Kim et al., 2008; Raaijmakers et al., 2010; Kode et al., 2014; Wang L. et al., 2014). As such, it has emerged as an attractive target for therapeutic modulation in hematological cancers such as myelodysplastic syndromes (MDS), chronic myelomonocytic leukemia (CMML), and acute myeloid leukemia (AML). This requires a deep understanding of the BM niche and its crosstalk with normal and malignant blood cells. Other direct clinical applications relate to allogeneic hematopoietic stem cell transplantation (HSCT), which remains the only curative option for many patients. Factors critical to its success include transfer of adequate numbers of HSCs but also their ability to engraft the recipient’s BM. Understanding the regulation and maintenance of HSCs by microenvironmental elements is therefore crucial for the development of strategies enabling the amplification of HSCs *ex vivo* before transplant, and for novel potential approaches to improve clinical engraftment through stromal cell co-transplantation.

In this review, we summarize advances made in the identification of BM microenvironmental components and their interactions with HSCs in health, physiological states and disease. We focus on the vascular elements of the BM HSC niche, which represents a dynamic interface spanning ontogenesis, through HSC regulation, to drug delivery. We discuss current and experimental approaches attempting to modulate these interactions in the clinic, with emphasis on myeloid malignancies.

## Localization of HSCs Within the BM Niche: Lessons From Mouse Models

**FIGURE 1:**
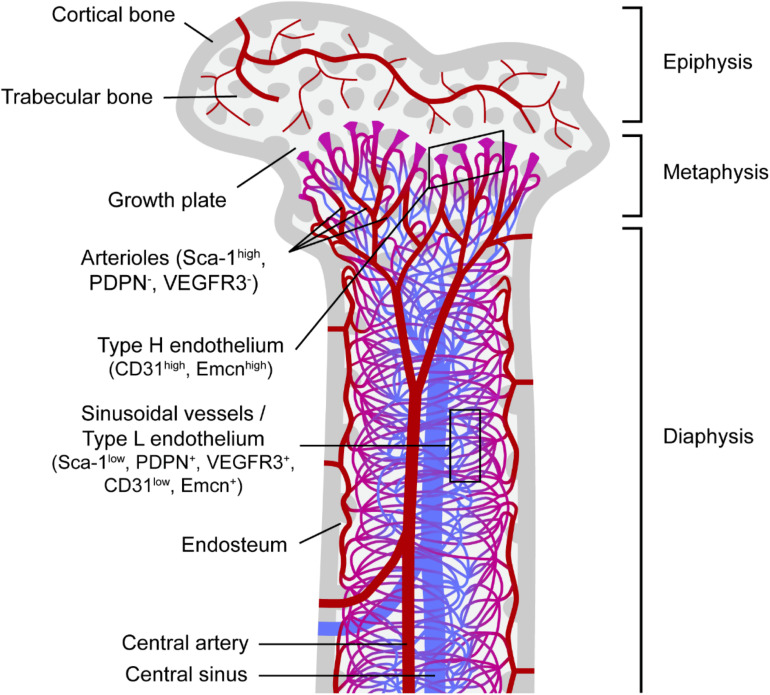
Bone marrow vascular network. Schematic representation of a femur section and the vascular component of the corresponding bone marrow. Trabecular bone, a type of porous bone with high turnover also called cancellous or spongy bone is mainly found at the ends of the femur in the epiphysis and metaphysis regions. The endosteum is known as the bone marrow region adjacent to bone. Columnar blood vessels corresponding to type H endothelium and arterioles are mainly found in the metaphysis, whereas sinusoidal vessels corresponding to type L endothelium are predominantly located in the diaphysis. Nutrients, oxygen and other factors enter the bone marrow mainly through the central artery, which branches into arterioles and then through transitional vessels into a vast network of sinusoids, fenestrated vessels where the exchange of cells and factors takes place. Sinusoids converge into the central sinus allowing for the exit of waste products from the BM through the venous circulation. Sca-1, stem cell antigen-1; PDPN, podoplanin; VEGFR3, vascular endothelial growth factor receptor 3; Emcn, endomucin.

The BM is a highly vascularized tissue found within medullary bone cavities and is the primary site of definitive hematopoiesis in vertebrates (Jagannathan-Bogdan and Zon, 2013). Oxygenated, nutrient-rich blood enters the BM through arteries that branch into thin-walled arterioles. Transitional vessels connect arterioles to vast networks of sinusoids: thin-walled fenestrated capillaries with wide lumen where exchange of cells and factors is highly facilitated. Webs of sinusoids converge into veins through which new blood cells egress BM and enter systemic circulation. In long bones (e.g., the femur) arterioles and transitional vessels are predominantly located near the endosteum (inner surface of the bone) or adjacent to trabecular bone, the spongy bone tissue with active remodeling being found mainly in the metaphysis (Figure 1).

Histological mapping of a cell population highly enriched in HSCs in BM sections was first achieved through application of signaling lymphocytic activation molecule (SLAM) family markers. CD150^+^CD48^–^CD41^–^Lineage^–^ HSCs were shown to localize close to sinusoids, with only a minority (<15%) residing in the endosteal region (Kiel et al., 2005). By contrast, visualization of HSCs following their isolation, labeling and transplantation into irradiated mice showed preferential localization of HSCs near the endosteum (Celso et al., 2009; Xie et al., 2009). However, BM irradiation can disrupt sinusoids (Hooper et al., 2009), raising the possibility that HSCs relocated to endosteal arterioles consequent upon destruction of sinusoids during conditioning. Transplanted HSCs were subsequently shown to preferentially locate near endosteal vessels even without prior BM ablation, and homing of transplanted hematopoietic stem and progenitor cells (HSPCs) was observed to preferentially occur in the metaphysis of femurs (rich in trabecular bone) in these non-irradiated mice (Ellis et al., 2011). Imaging advances permitted three-dimensional visualization of HSCs also using SLAM family markers, which revealed the association of quiescent HSCs with small arterioles, highly abundant in the endosteum, with HSCs migrating away from the proximity to arterioles upon activation (Kunisaki et al., 2013).

The development of a HSC reporter mouse through knock-in of green fluorescent protein (GFP) in the *Ctnnal1* gene (α-*catulin*^*GFP*^) later enabled deep imaging of optically-cleared BM in combination with immunostaining for one additional marker (c-Kit) for the identification of HSCs (Acar et al., 2015). This study showed that α*-catulin*-GFP^+^ c-Kit^+^ cells were more abundant in central marrow than in proximity to bone surfaces, being predominant in the diaphysis versus the metaphysis (Figure 1). Notably, 84% of HSCs were located within 10 μm of a sinusoidal blood vessel; however, this was no different from the distribution of random dots, reflecting the high density of sinusoids in central BM. Independently of proliferation status, most HSCs were located distant from bone surfaces, arterioles and transitional vessels (Acar et al., 2015). Subsequent imaging of chemically-cleared BM in another HSC reporter murine model based on expression of *Hoxb5*, which in BM is limited to long-term HSCs (LT-HSCs), reinforced the idea of a perivascular HSC niche by showing that > 94% Hoxb5-mCherry^+^ LT-HSCs associated with VE-cadherin^+^ endothelial cells (ECs) (Chen et al., 2016). This was significantly higher (∼50%) than expected for a random distribution of dots, although no distinction between different vascular compartments was made.

Recently, the development of a highly-specific HSC reporter mouse permitted *in vivo* imaging of HSCs in their natural environment without requirement for additional markers or clearing techniques (Christodoulou et al., 2020) that can themselves perturb the BM niche (Richardson and Lichtman, 2015). This model began with generation of a myelodysplastic syndrome 1 (*Mds1*) gene reporter mouse (*Mds1*^*GFP/*+^), where GFP expression is largely restricted to a mixed population of HSPCs. Exploiting the fact that *Flt3* is expressed in early hematopoietic progenitors but not HSCs, crossing of *Mds1*^*GFP/*+^ mice (with loxP sites flanking the GFP coding sequence) with a *Flt3*-Cre strain allowed for disruption of GFP expression in hematopoietic progenitors (*Flt3* expressing cells), thereby restricting the GFP^+^ population to LT-HSCs (Christodoulou et al., 2020). Live-imaging of the calvaria in these mice showed that both GFP^+^ HSPCs in *Mds1*^*GFP/*+^ mice and GFP^+^ LT-HSCs in *Mds1*^*GFP/*+^*Flt3*^*Cre*^ mice locate near blood vessels, with an average distance to the closest vessel under 10 μm (Christodoulou et al., 2020). However, while GFP^+^ LT-HSCs in the *Mds1*^*GFP/*+^*Flt3*^*Cre*^ mice associated almost exclusively with sinusoids, GFP^+^ HSPCs in *Mds1*^*GFP/*+^ mice were also found close to transition zone vessels. Furthermore, while GFP^+^ LT-HSCs in *Mds1*^*GFP/*+^*Flt3*^*Cre*^ mice localized near endosteum, GFP^+^ HSPCs in *Mds1*^*GFP/*+^ mice showed more variable distance from endosteum (Christodoulou et al., 2020). This suggests a potential dual endosteal-perivascular HSC niche, as had been proposed in other studies (Celso et al., 2009; Kunisaki et al., 2013; Nombela-Arrieta et al., 2013), and supports the existence of distinct niches for HSCs and hematopoietic progenitors.

This study also investigated the localization of HSCs following activation induced by cyclophosphamide/granulocyte colony-stimulating factor (Cy/GCSF) or 5-fluorouracil (5-FU) treatment. Despite a heterogeneous response, GFP^+^ LT-HSCs in *Mds1*^*GFP/*+^*Flt3*^*Cre*^ mice, which displayed low motility in steady-state, generally increased motility following treatment, moving further from endosteum and closer to the vasculature, with the average distance to the closest vessel reducing to ∼1 μm and the preferential association with sinusoids preserved (Christodoulou et al., 2020). Interestingly, clonal expansion of activated HSCs reportedly took place mainly in BM cavities with active bone-remodeling, although the potential mechanisms favoring HSC expansion in these specific locations remain unknown.

Most recently, three existing reporter models [α*-catulin^*GFP/*^*^+^ (Acar et al., 2015); *Mds1*^*GFP/*+^*Flt3*^*Cre*^ (Christodoulou et al., 2020)*; double transgenic SCL-tTA; H2B-GFP* (Wilson et al., 2008)] were brought together for a comprehensive and extensive quantification of HSC localization relative to nine different components of mouse BM, at the tissue-wide level and with single-cell resolution (Kokkaliaris et al., 2020). This concluded that while femoral adult HSCs are mostly found close to sinusoids, Cxcl12 stroma and megakaryocytes, this does not represent an active enrichment but instead simply reflects higher abundance of these BM components. Nevertheless, this does not exclude the existence of key contributions from specific BM components to the maintenance of HSCs through secreted factors, an unrevealed heterogeneity in BM populations, or an induction of HSC-supportive function in specific BM components following their interaction with HSCs (Silberstein et al., 2016).

## Cellular Components of the BM Niche and Their Interactions With Hematopoiesis

### Osteoblasts

The suggestion in early studies that HSPCs might be enriched in endosteal areas where osteoblasts are highly abundant (Lord et al., 1975; Gong, 1978), and the ability of osteoblasts to maintain hematopoietic progenitors *in vitro* (Taichman et al., 1996), first hinted that these might form a component of the HSC BM niche. Mouse models with increased number of osteoblasts generated through overexpression of parathyroid hormone or deletion of *Bmpr1a* showed higher numbers of cells in a population highly enriched in HSPCs (Calvi et al., 2003; Zhang et al., 2003). Furthermore, conditional ablation of osteoblasts led to a decrease in the number of myeloid, lymphoid and erythroid progenitors, as well as HSCs (Visnjic et al., 2004). However, while these studies supported a role for osteoblasts in the maintenance of HSPCs in the BM niche, no direct causal mechanism was confirmed.

Chemokine (C-X-C motif) ligand 12 (CXCL12) and stem cell factor (SCF; KIT-ligand) are the two cytokines whose role in HSC maintenance has been most reproducibly shown, exerting their function through binding to chemokine (C-X-C motif) receptor 4 (CXCR4) and KIT, respectively, on HSCs. Both CXCL12 and SCF support quiescence in HSCs, and CXCL12 also plays a role directing HSCs to and retaining them within BM (Ogawa et al., 1991; Petit et al., 2002; Ara et al., 2003; Sugiyama et al., 2006; Thorén et al., 2008). Expression of these factors by osteoblasts implied that these cells might mediate regulation of HSCs. However, deletion of CXCL12 or SCF in osteoblasts in mouse models had no significant effect on HSCs (Ding et al., 2012; Ding and Morrison, 2013; Greenbaum et al., 2013), suggesting against any direct HSC-regulatory role. Potential regulation instead through osteoblast production of other factors previously associated with HSC maintenance, including osteopontin (OPN) (Nilsson et al., 2005; Stier et al., 2005), thrombopoietin (TPO) (Qian et al., 2007; Yoshihara et al., 2007), and angiopoietin 1 (ANGPT1) (Arai et al., 2004) has been investigated: but not confirmed for OPN, and excluded for TPO and ANGPT1 (Zhou et al., 2015; Decker et al., 2018).

Finally, despite several studies reporting localization of HSCs in close proximity to endosteum, three-dimensional imaging has not revealed a significant association between endogenous HSCs and osteoblasts (Kunisaki et al., 2013; Nombela-Arrieta et al., 2013). It therefore remains unclear whether osteoblasts play a direct role in the regulation of HSCs. However, other lines of evidence support a function as regulators of other hematopoietic progenitors (Visnjic et al., 2004; Zhu et al., 2007; Ding and Morrison, 2013; Greenbaum et al., 2013; Silberstein et al., 2016).

### Endothelial Cells

Hematopoietic stem cells and ECs share a common derivation route, through the process of “endothelial-to-hematopoietic transition” (Ottersbach, 2019). As such, the hematopoietic and vascular systems are fundamentally and intrinsically linked. In support, hematopoietic and EC gene expression profiles show considerable overlap, whilst ECs share similar adhesion molecules and genetic abnormalities with malignant myeloid cells (Teofili et al., 2011; Issa, 2013).

Links between hematopoietic and ECs go beyond the ontogenic and molecular, to the functional. The potential of ECs as HSC regulators was first inferred from *in vitro* experiments in which ECs isolated from the yolk sac or aorta-gonad-mesonephros region were shown to support HSC maintenance and expansion (Cardier and Barberá-Guillem, 1997; Ohneda et al., 1998; Li et al., 2004). Since then, the repeated association of HSCs with blood vessels in studies of HSC localization has motivated extensive investigation into the contribution of ECs to HSC regulation. Since reports detailing the type of blood vessels with which HSCs associate have been inconclusive, studies on the HSC-supportive potential of ECs have typically distinguished between distinct types of endothelium, and provided evidence supporting the different results obtained from HSC localization studies. Primarily, arterial/arteriolar BM ECs and sinusoidal BM ECs have been distinguished through their differential expression of Sca-1 (high in arteriolar; low in sinusoidal), either alone or in combination with other markers such as podoplanin (PDPN) or vascular endothelial growth factor receptor 3 (VEGFR3), which are expressed almost exclusively in sinusoidal ECs (Hooper et al., 2009; Kunisaki et al., 2013). Additionally, a recent study defined the so-called type H endothelium [high expression of CD31 and endomucin (Emcn)], which most likely corresponds to transitional vessels, and type L endothelium (low CD31/Emcn) corresponding to sinusoidal vessels (Kusumbe et al., 2014).

Among the first studies confirming the role of ECs in HSC regulation, conditional deletion of *Scf* and *Cxcl12* using the pan-endothelial-specific *Tie2-Cre* mouse strain was shown to compromise HSC maintenance (Ding et al., 2012; Ding and Morrison, 2013; Greenbaum et al., 2013). Subsequent studies then reported higher expression of SCF in arteries and type H ECs compared to type L ECs (Kusumbe et al., 2016); and deletion of *Scf* in arteriolar, but not in sinusoidal, ECs led to impairment of HSC regeneration capacity (Xu et al., 2018). Moreover, single cell RNA-seq analyses recently confirmed higher expression of *Scf* and *Cxcl12* in arteriolar BM ECs (Baryawno et al., 2019; Tikhonova et al., 2019). Taken together, these studies support the role of arteriolar and potentially transitional vessels as HSC regulators. Accordingly, the lower permeability of arterioles has been proposed to result in lower concentration of reactive oxygen species (ROS) in neighboring HSCs, thereby conferring a quiescent phenotype, while higher production of ROS in HSCs surrounding sinusoids has been associated with their differentiation and migration into blood (Itkin et al., 2016).

Conversely, the higher level of hypoxia found in the perisinusoidal central areas of BM compared to the endosteal region rich in small arteries (Spencer et al., 2014), the association of hypoxia with HSC quiescence (Hermitte et al., 2006; Eliasson et al., 2010) and previous studies suggesting that HSC localization might be determined by areas with lowest oxygen tension (Parmar et al., 2007), alternatively support a perisinusoidal HSC niche. However, this was recently challenged by studies using the *Mds1*^*GFP/*+^ and *Mds1*^*GFP/*+^*Flt3*^*Cre*^ mouse models, in which measurement of local pO_2_ surrounding GFP^+^ HSPCs in *Mds1*^*GFP/*+^ mice and GFP^+^ LT-HSCs in *Mds1*^*GFP/*+^*Flt3*^*Cre*^ mice revealed similar oxygen levels, and neither GFP^+^ HSPCs nor GFP^+^ LT-HSCs localized in the BM regions with deepest hypoxia (Christodoulou et al., 2020).

Relevant to the mechanism of HSC regulation by ECs is their expression of Notch ligands. Conditional deletion of *Jagged1* (*Jag1*) in ECs using the pan-endothelial-specific *Cdh5-Cre* mouse strain impaired HSC self-renewal and regeneration capacity (Poulos et al., 2013). Additionally, activation of Notch signaling in ECs through inactivation of *Fbxw7*, which is required for the degradation of Notch, increased BM HSC numbers; whilst inactivation of the Notch ligand *Dll4* in ECs reduced the repopulation potential of BM cells (Kusumbe et al., 2016). In accordance with the previously reported role of Notch signaling in expansion of arterioles and type H endothelium (Ramasamy et al., 2014), EC-specific Notch activation also resulted in increased abundance of arterioles, Sca1^+^ and ephrin-B2^+^ ECs (corresponding to arteriolar ECs), PDGFRβ^+^ perivascular cells (described later) and SCF levels, whilst inactivation of *Dll4*, or of the downstream DNA-binding protein Rbpj, induced the opposite effects (Kusumbe et al., 2016). Therefore, the observed changes in HSC frequencies when manipulating Notch signaling are most likely the consequence of changes in blood vessels rather than a direct regulatory effect on HSCs themselves.

In addition to Notch, hypoxia-inducible factor (HIF) signaling has also been shown to promote the expansion of type H ECs (Kusumbe et al., 2014). Moreover, EC-specific HIF-1α mutants present lower numbers of type H ECs, Sca1^+^ ECs, ephrin-B2^+^ ECs, PDGFRβ^+^ perivascular cells, lower SCF levels and decreased HSC frequency (Kusumbe et al., 2016). However, despite expanding numbers of type H ECs, inactivation of the von Hippel–Lindau (VHL) protein, which mediates HIF-1α degradation, did not lead to an increase in arterioles, PDGFRβ^+^ perivascular cells, HSC frequencies or cellular SCF levels (although it did increase secreted SCF) (Kusumbe et al., 2016). This highlights an essential role of Notch signaling in enhancing HSC numbers through expansion of functional vasculature (Kusumbe et al., 2016). However, some studies reporting a requirement for Notch signaling for HSC maintenance have recently been challenged. For example, EC-specific deletion of *Dll4* (which in previous studies resulted in decreased HSC numbers) was since shown to alter the proportions of hematopoietic progenitors, leading to BM myeloid skewing, but not overall HSC frequencies (Tikhonova et al., 2019). These discrepancies could be explained by differences in timing of induction of *Dll4* deletion.

Hematopoietic cells themselves have further been shown to be required for the maintenance of the BM vasculature, with hematopoietic cell ablation in a *Vav1-Cre Rosa26*-DTR mouse model leading to multiple vascular alterations (Chen et al., 2019). In this study, HSPCs were reported to target a rare subset of apelin(Apln)-expressing BM ECs through secretion of VEGF-A, a mechanism underlying vascular normalization after EC injury by myeloablative chemotherapy or irradiation. Genetic inactivation of VEGFR2 in Apln^+^ ECs also decreased HSC percentages, suggesting a role for this EC subpopulation in the maintenance of HSCs in steady-state dependent on VEGF-signaling, which might be (at least partially) activated by VEGF-A secreted by hematopoietic cells (Chen et al., 2019). In the opposite direction, EC-derived VEGF-C (another ligand for VEGFR2 that also binds VEGFR3) is important for HSCs. Loss of EC-derived VEGF-C was reported to impair expression of key HSC-supporting factors both in ECs and in associated Lepr^+^ perivascular cells (described later), with these effects being dependent on VEGF-signaling regulation in ECs (Fang et al., 2020).

Finally, NF-κB signaling and expression of the cytokine receptor subunit gp130 and adhesion molecule E-selectin in ECs have also been shown to play roles in HSC regulation (Yao et al., 2005; Winkler et al., 2012; Poulos et al., 2016). Interestingly, the expression of gp130, reported to support hematopoiesis, and E-selectin, associated with negative regulation of HSC quiescence and self-renewal, were recently shown to be restricted to sinusoidal ECs (Baryawno et al., 2019; Tikhonova et al., 2019).

### Perivascular MSCs

Mesenchymal stem cells (MSCs) are rare multipotent cells found within BM stroma with capacity to differentiate into many cell types, including osteoblasts, adipocytes and chondrocytes (Dominici et al., 2006). Mesenchymal stem or early progenitor cells play key roles in HSC regulation, although identification of specific subpopulations with fundamental niche functions still lacks specificity or consensus.

The generation of a Cxcl12 reporter mouse model through knock-in of GFP into the *Cxcl12* locus first led to the identification of perivascular stromal cells, with high expression of Cxcl12 and potential to differentiate into adipogenic and osteogenic lineages, and whose depletion reduced HSC numbers (Sugiyama et al., 2006; Omatsu et al., 2010). These cells, denominated “Cxcl12-abundant reticular” (CAR) cells, localized mainly surrounding sinusoids and represent major Cxcl12 and SCF producers in BM (Sugiyama et al., 2006; Omatsu et al., 2010). Additionally, perivascular MSCs identified through Nestin expression in a *Nestin*-GFP transgenic mouse model co-localized with HSCs and expressed high levels of Cxcl12 and SCF. Ablation of these Nestin^+^ cells in a *Nestin-Cre^*ERT2*^*/iDTR mouse model resulted in decreased HSC numbers (Méndez-Ferrer et al., 2010). Isolation of a largely overlapping population was later identified through co-expression of both PDGFRα and CD51 (Pinho et al., 2013).

A *Scf*^*GFP*^ knock-in reporter mouse model found the highest expression of SCF throughout all BM in perivascular cells, and identified a perivascular SCF^+^ population with mesenchymal features and expression of leptin receptor (*Lepr*) (Ding et al., 2012). Around 90% overlap was observed between Lepr-Cre^+^ and CAR cells (Zhou et al., 2014), and Lepr-Cre^+^ cells were determined to be a subpopulation (constituting ∼80%) of Nestin-GFP^+^ cells (Kunisaki et al., 2013; Mizoguchi et al., 2014). Subsequent studies further highlighted heterogeneity of the Nestin-GFP^+^ population, showing that the Lepr^+^ subpopulation, which is Nestin-GFP^*low*^ with high levels of Cxcl12 and SCF, associated with sinusoids, whereas a Nestin-GFP^*high*^ subpopulation positive for neural/glial antigen 2 (Ng2) and expressing high levels of Cxcl12 localized peri-arteriolarly (Kunisaki et al., 2013; Zhou et al., 2014; Asada et al., 2017).

Conditional deletion of Cxcl12 in *Ng2-Cre* mice dramatically reduced HSC numbers in BM and promoted their exit from quiescence and migration away from arterioles (Asada et al., 2017). However, conditional *Cxcl12* deletion in *Lepr-Cre* mice mobilized HSC egress, but did not significantly alter their observed frequency in the BM (Ding and Morrison, 2013; Asada et al., 2017). Conversely, conditional deletion of *Scf* depleted BM HSCs in *Lepr-Cre* mice, but did not significantly alter HSC numbers in *Ng2-Cre* mice (Ding et al., 2012; Asada et al., 2017). These results further support the heterogeneity of the perivascular BM niche, with different types of mesenchymal stem and progenitor cells residing in different locations and supporting HSC maintenance through different cytokine contributions.

The precise identities, distinctions, and overlaps between described MSC populations remain somewhat unclear. Conditional deletion of *Cxcl12* or *SCF* in *Nestin-Cre* mice had no significant effect on HSC numbers (Ding et al., 2012; Ding and Morrison, 2013), which does not correlate well with the reported high degree of overlap between the Nestin-GFP^+^ and Lepr^+^ populations (Kunisaki et al., 2013; Mizoguchi et al., 2014). *Nestin*-*GFP* and *Nestin-Cre* mouse models have been shown to mark different cell populations, with Nestin-GFP^+^ cells not well representing the level of endogenous Nestin expression (Zhou et al., 2014). The *Nestin-GFP* and *Nestin-Cre*^*ERT2*^ mouse models have been shown to label not only MSCs but also ECs (Ding et al., 2012; Ono et al., 2014).

Recent single cell RNA-seq studies have facilitated highly refined transcriptional characterization of BM stromal subpopulations, improving definitions of distinct mesenchymal populations and their differentiation paths. A predominant BM MSC population has been defined as Lepr^+^ and shown to have pre-adipocytic features, with high expression of Cxcl12, SCF and Angiopoietin-1 (Baryawno et al., 2019). No Nestin or NG2 expression was detected in this population, challenging the previously reported high overlap between Lepr^+^ and Nestin^+^ populations; also recently called into question by a single-cell protein expression study that identified an overlap of just ∼3% between Lepr- and Nestin-expressing cells (Severe et al., 2019). Within Lepr^–^ MSCs four clusters have been distinguished, differing in gene expression patterns which alternatively presented adipogenesis- or osteogenesis-related markers and in some cases differential expression of HSC regulators (Baryawno et al., 2019; Tikhonova et al., 2019). Additionally, a mixed population of BM MSCs and pericytes (contractile cells also surrounding vessels) co-expressing Nestin, NG2 and the pericyte markers Acta2, Myh11, and Mcam, has also been described (Baryawno et al., 2019). This population, which expressed very low levels of Lepr, could be further subdivided into distinct clusters with different expression patterns of Lepr, Nestin, and NG2, as well as Cxcl12 and SCF. Most recently, another study focused on the differentiation trajectories of MSCs into osteoblasts, adipocytes and chondrocytes distinguished seven BM mesenchymal subpopulations, ranging from MSCs through distinct early and late progenitors (Wolock et al., 2019). Thus, the BM mesenchymal niche is revealing hitherto underappreciated complexity and has thus far eluded robust and exhaustive characterization. Functional studies are required to fully determine the specific contribution of each subpopulation to HSC regulation.

### Adipocytes

The proportion of adipocytes in human BM progressively increases with age, leading to replacement of “red” BM, comprising high hematopoietic activity, by fatty “yellow” BM, which constitutes around 70% of BM space by area in adults and has reduced hematopoietic potential (Kricun, 1985; Ambrosi et al., 2017). Additionally, post-irradiation BM engraftment was improved in a “fatless” mouse model lacking adipocytes or following treatment with an adipogenesis inhibitor (Naveiras et al., 2009; Zhu et al., 2013). Together these suggest a negative effect of adipocytes on HSC function. Conversely, it has also been shown that adipocytes, together with Lepr^+^ MSCs, become a primary source of SCF following irradiation promoting hematopoietic regeneration (Zhou et al., 2017). Further studies are required to clarify the role of adipocytes in both steady state and regenerative regulation of HSCs.

### Neuronal Cells

Sympathetic nerves penetrating the BM regulate HSC mobilization into circulation in a circadian manner (Katayama et al., 2006; Méndez-Ferrer et al., 2008). Noradrenaline is the main neurotransmitter released by sympathetic fibers, signaling through adrenergic receptors. Sympathetic fibers innervating BM induce circadian fluctuations in Cxcl12 expression in perivascular MSCs, which express the β_3_-adrenergic receptor, resulting in oscillations of HSC egress from BM (Méndez-Ferrer et al., 2008, 2010). Furthermore, the sympathetic nervous system mediates HSC mobilization in response to granulocyte colony-stimulating factor (G-CSF) (Katayama et al., 2006), and plays a role in hematopoietic regeneration after injury (Lucas et al., 2013; Park et al., 2015). Additionally, Schwann cells (myelinated glial cells of the peripheral nervous system), have been reported to promote HSC quiescence through activation of transforming growth factor beta (TGF-β) (Yamazaki et al., 2011).

### Hematopoietic Cells

In addition to the BM stromal cell types discussed above, some hematopoietic lineages themselves derived from HSCs have been reported to contribute to HSC regulation. Among these, megakaryocytes were shown to induce quiescence in HSCs (Bruns et al., 2014; Nakamura-Ishizu et al., 2014; Zhao et al., 2014), and regulatory T cells to promote survival of transplanted allogenic HSCs (Fujisaki et al., 2011). The importance of macrophages in HSC homeostasis has been elegantly demonstrated in a series of experiments in which macrophage depletion in two models, (depletion of phagocytes with clodronate-loaded liposomes in wild type mice and ablation of *c-fms*–expressing cells in Mafia transgenic mice) was shown to enhance HSPC mobilization into the bloodstream (Winkler et al., 2010; Chow et al., 2011). BM macrophages are key to the process of clearing CD62L^*low*^CXCR4^*high*^ aged neutrophils by phagocytosis, which leads to the generation of liver X receptor (LXR)-dependent homeostatic signals, resulting in reductions in the size and function of the hematopoietic niche *via* the circadian egress of HSPC into circulation (Casanova-Acebes et al., 2013).

## Role of the BM Vascular Niche in Physiological Processes

### Aging

Hematopoietic stem cell function, dependent on both cell-intrinsic and extrinsic cues, undergoes progressive decline during the normal aging of an organism (Morrison et al., 1996; Rossi et al., 2005; Ergen et al., 2012; Geiger et al., 2013; Beerman and Rossi, 2014). Cell-intrinsic changes in cell polarity, epigenetic landscape, and genomic integrity that accompany aging are major drivers for altered HSC function, but factors contributed by the aged BM microenvironment also play important roles (Rossi et al., 2005; Ho and Méndez-Ferrer, 2019). Age-related changes in the composition (e.g., decrease in bone formation; altered extracellular matrix components; increase in adipogenesis; increased number and altered function of homeostatic macrophages and megakaryocytes), proliferative capacity, spatial arrangement, adhesion molecule expression and secretome of niche cells are all observed features that potentially influence the natural temporal decline in HSC function (Köhler et al., 2009; Florian et al., 2012; Maryanovich et al., 2018; Ho et al., 2019). This is well illustrated by the interaction between macrophages and HSCs in aging. Frisch et al. (2019) demonstrated that aged C57BL/6J.NIA mice BM macrophages were deficient in their ability to engulf senescent neutrophils, which in turn led to increased IL-1β levels. Aged macrophages were also determined to have a pro-inflammatory phenotype demonstrating upregulation of *Il1b*, resulting in HSC platelet skewing (Frisch et al., 2019).

#### Vascular Niches in Aging

Using time-lapse 2-photon microscopy Hartmut Geiger’s group first showed that HSPCs from aged mice displayed distinct relationships to BM architectural features (including more distant localization from endosteum), as well as decreased adhesion to stroma, compared with HSPCs in younger animals (Köhler et al., 2009). They subsequently provided empirical evidence that microenvironmental components directly impose fitness constraints on HSCs, demonstrating that older HSCs showed increased myelomonocytic skewing, a cardinal feature of aged hematopoiesis, when transplanted into young recipient mice. Conversely, myeloid output from young HSCs was relatively augmented following transplantation into older recipients (Vas et al., 2012). Moreover, the BM microenvironment of older mice promoted oligoclonal hematopoietic expansion (Vas et al., 2012), another feature observed with aging and one that represents a preleukemic clonal restriction step. Recently, transplantation of HSCs from old mice into young recipients (without irradiation) reprogrammed the transcriptome of HSCs harvested from secondary recipients to resemble that of young HSCs, although only partly restored functional defects in this model (Kuribayashi et al., 2019).

During physiological aging, BM ECs gradually lose their ability to maintain HSC homeostasis (Ramalingam et al., 2017). Using an *ex vivo* HSPC/EC co-culture system followed by *in vivo* murine transplantation, Poulos et al. showed that HSPCs co-cultured with aged ECs displayed dramatic reduction in repopulation capacity in comparison with young ECs. Infusion of ECs following myeloablation promoted hematopoietic recovery; however, aged ECs lost this regenerative potential, indicating a detrimental effect of aging on their hematopoietic supportive capacity (Poulos et al., 2017). In contrast, young ECs could restore regenerative capacity of aged HSCs, albeit failing to correct the myeloid bias of older HSCs. Aged ECs have also been shown to express lower levels of SCF, Cxcl12, and Notch ligands Dll4 and Jag2. Since conditional deletion of these factors in ECs also results in reduced HSC numbers in murine models (Ding et al., 2012; Greenbaum et al., 2013; Poulos et al., 2013), reduction in expression of these critical factors might be directly implicated in the age-related drop in their potential to support HSCs. Given the extensive cross-talk between niche cells it remains to be determined whether reduced expression of these factors affects HSC homeostasis directly, or indirectly through altering function of other cells within the niche (Pinho and Frenette, 2019). The aged BM microenvironment also shows a decrease in mTOR signaling, which in ECs is important for HSC maintenance. Indeed, specific inhibition of mTOR signaling in ECs in young mice resulted in an aged HSPC phenotype (Ramalingam et al., 2020).

Advanced imaging has revealed substantial BM vascular remodeling associated with aging. Aged mice show a relative decline in CD31^*high*^Emcn^*high*^ type H endosteal ECs, whereas sinusoidal type L ECs (CD31^*low*^Emcn^*low*^) remain unchanged (Kusumbe et al., 2016). Reduction in type H ECs is linked to defects in osteogenesis and angiogenesis (Kusumbe et al., 2014), although how this relates to altered HSC function remains uncertain. As discussed earlier, the Notch pathway plays a key role in maintenance of endosteal niches by regulating the formation and abundance of arterioles and type H ECs (Ramasamy et al., 2014; Kusumbe et al., 2016). In contrast, Notch activation in ECs induces elevated SCF and increased HSC frequency. Conditional deletion of the Notch ligand *Dll4* in ECs caused decrease in HSC self-renewal and promoted expansion of myeloid progenitors at the expense of lymphoid progenitors, a cardinal feature of aged-HSCs (Kusumbe et al., 2016). Notch reactivation specifically in ECs reversed the age-related decrease in type H capillaries and induced osteogenesis and angiogenesis (Ramasamy et al., 2016). Thus, altered Notch signaling critically contributes to the vascular niche mediating age-related HSC dysfunction.

#### Neurovascular Niches in Aging

Another recent study observed contraction of HSC-supporting endosteal niches, with expansion of non-endosteal neurovascular HSC niches, in models of both physiological and pathological premature aging (Ho et al., 2019). These neurovascular BM niches were marked by increased noradrenergic nerve fibers in aged mice. Increased sympathetic activity caused myeloid skewing of aged HSCs through predominant activation of β2-adrenergic receptors (AR), promoting IL-6-dependent megakaryopoiesis; deletion of β2-AR in stromal cells halved myeloid and megakaryocyte lineage cells in adult mice (Ho et al., 2019). Interestingly, β3-AR activation exhibited the opposite effect on lympho-myeloid bias, suggesting that lack of stromal β3-AR might accelerate HSC aging, and that a switch toward predominant β2-AR activation might be implicated in age-related HSC dysfunction (Ho et al., 2019). Whether and to what degree age-related hematopoietic dysfunction is reversible remains unknown, but could have transformative clinical impact. Since many changes associated with aging, including those affecting vascular niche components, are rooted in epigenetic and/or signaling changes, it is plausible that these could be modulated and their consequences reversed. This raises the prospect of potentially restoring HSC fitness and function and, given the connection between genetic loci that link aging phenotype in HSCs and lifespan, could even rejuvenate entire organisms (Geiger et al., 2001; Li et al., 2020). Aged BM stroma expresses low levels of OPN and exposure of young HSCs to an OPN^*ko*^ niche recapitulated features of age-related HSC dysfunction, directly implicating microenvironmental OPN loss in promoting HSC aging (Guidi et al., 2017). Intriguingly, treatment of old HSCs with thrombin-cleaved OPN reversed these features, suggesting a potential novel treatment to ameliorate HSC aging. Another approach could involve administration of β3-AR agonists, to revert the balance of sympathetic nerve signaling away from age-related predominant β2-AR activation (Ho et al., 2019). In support, treatment of old mice with the β3-AR-selective sympathomimetic BRL37344 markedly recovered HSC engraftment potential and multilineage contribution in serial competitive transplantation experiments (Maryanovich et al., 2018); the same agonist effected comparable HSC rejuvenation in a murine model of premature aging (Ho et al., 2019), and blocked disease progression in a JAK2-mutant myeloproliferative neoplasm model (Arranz et al., 2014).

### Inflammation

Chronic inflammation is a feature of the aged BM microenvironment and the role of “inflamm-aging” on HSC function is well described (Kovtonyuk et al., 2016). Chronic inflammation in ECs impairs their ability to support HSC homeostasis (Denkinger et al., 2015). Under inflammatory conditions, ECs produce G-CSF, promoting the myeloid lineage bias typical of aged hematopoiesis (Poussin et al., 2014). Activation of inflammation in BM ECs with TNF-α induced GM-CSF-mediated proliferation and exhaustion of HSPCs in a Notch-dependent manner (Fernandez et al., 2008). Conditional deletion of *RBPJ* induced up-regulation of *miR-155* resulting in constitutive activation of NF-κB signaling *via* down-regulation of the NF-κB inhibitor kB-ras1. The increase in expression of proinflammatory cytokines (including TNF-α) led to development of a myeloproliferative neoplasm in these KO mouse models (Wang L. et al., 2014). *miR-155* levels are elevated in total BM cells of myelofibrosis patients compared with controls suggesting the role of notch signaling and inflammation in ECs in development of hematological disorders. Indeed, following myelosuppression, targeting inflammation by blocking the NF-κB pathway in ECs increased HSC self-renewal and regenerative capacity (Poulos et al., 2016).

The aforementioned study of microenvironmental aging by Ho et al. (2019) reported age-related increases in BM concentrations of IL-1α, IL-1β, and IL-6: inflammatory cytokines that drive expansion of myeloid and megakaryocytic cells (Pietras, 2017) which are themselves a major source of inflammatory cytokines (Pietras, 2017), potentially establishing a positive feedback loop to exacerbate inflammatory vascular remodeling and the myeloid skewing observed in aging BM. An inflammatory niche not only suppresses normal hematopoietic function but also promotes mutant HSC expansion. Consistent with this, *TET2*-mutant human HSPCs are resistant to apoptosis and display greater fitness under inflammatory microenvironment conditions (Abegunde et al., 2018). Future studies should also explore the complex interaction between inflammatory cells, such as macrophages (Seyfried et al., 2020), and BM niches. Although macrophages are known pro-angiogenic cells (Polverini et al., 1977) that mediate vascular anastomosis (Fantin et al., 2010) and endothelial pruning in vascular development (Du Cheyne et al., 2020), the specific interaction between BM macrophages and vascular niches in the regulation of HSCs remains underexplored.

### Clonal Hematopoiesis

In recent years the concept of clonal hematopoiesis of indeterminate potential (CHIP) has emerged as a common age-related phenomenon (Steensma et al., 2015). It is characterized by presence of distinct hematopoietic subclones displaying genetic abnormalities characteristic of myeloid malignancies, but without evidence of overt hematological disease. Mutations are dominated by epigenetic modifier genes (DNMT3A; TET2; and ASXL1) and typically present at low levels, but by definition at ≥ 2% variant allele frequency (Steensma et al., 2015). CHIP is a potentially “pre-leukemic” state, although < 1% progress to develop frank hematological malignancy per year. CHIP has, however, been clearly linked to increased all-cause mortality, largely through an increased cardiovascular risk of up to ∼40-fold (Jaiswal et al., 2017; Libby et al., 2019). Incidence of CHIP is intimately linked to age, being found in <1% of individuals below 50 years but rising sharply to >20% of individuals in their tenth decade (Genovese et al., 2014; Jaiswal et al., 2014).

Whilst several cell-intrinsic factors mediate the risk of CHIP progressing to frank malignancy (e.g., the nature, identity and VAF of mutations involved) aging of the BM microenvironment also influences its natural history, promoting the expansion and evolution of these pre-malignant clones. Indeed, mathematical models suggest that non-cell autonomous features rather than somatic mutations predominantly drive clonal expansion of mutated stem cells (Rozhok et al., 2014). This is further supported by the presence of multiple CHIP clones (carrying SF3B1 and SRSF2) within the same individual (McKerrell et al., 2015). Vas et al. recently reported compelling evidence that the aged BM microenvironment directly exerts selection pressure on dominant HSPC clones generated to have intrinsic potential for clonal expansion using a retroviral insertional mutagenesis approach (Vas et al., 2012). In this well controlled model, dominant HSPCs remained predominantly oligoclonal when transplanted into young mice, whereas identical pools of cells transplanted into BM of older animals resulted in substantially reduced comparative clonality. Thus, exposure to aged BM microenvironment was apparently sufficient to promote transition to monoclonality, thus potentially mediating the progression of CHIP toward leukemia initiation.

Precisely what molecular mechanisms, and which features of the aged niche are implicated, remain unknown. Given the emerging links between inflammation and leukemia initiation (Pietras, 2017; Craver et al., 2018) it is tempting to speculate around the role of “inflamm-aging” of ECs in CHIP and its progression to leukemia, although direct empirical proof is currently lacking.

## BM Vascular Niche in Myeloid Malignancies

### Acute Myeloid Leukemia

Multiple studies have highlighted the importance of the microenvironment, and particularly the BM vasculature, in leukemia initiation, progression and chemoresistance (Duarte et al., 2018b). AML is a clonal hematopoietic malignancy characterized by uncontrolled proliferation of immature myeloid precursors (“blasts”) that accumulate in BM and blood, associated with progressive failure of normal hematopoiesis. AML represents the malignant transformation of HSCs or early myeloid progenitors, with a subpopulation of apical “leukemia stem cells” sustaining the disease, driving chemoresistance and effecting disease relapse, the main impediment to long-term survival (Thomas and Majeti, 2017). Increasing evidence implicates hijacking of the vascular niche by leukemic blasts, which take advantage of the signals regulating proliferation, differentiation and quiescence of HSCs in the healthy setting to promote their own proliferation, limit their differentiation and induce or maintain their stem cell-like properties, eventually displacing normal HSCs from their niches (Cogle et al., 2016; Hira et al., 2017; Kumar and Chen, 2018).

#### AML-EC Crosstalk

Acute myeloid leukemia cells share phenotypic and functional features with ECs, reflecting a close relationship and their common ontogeny. Notably, CD34, the archetypal surface marker of HSPCs and most AML blasts, is expressed outside hematopoiesis exclusively on endothelium. AML blasts variably express members of the VEGF family, the most potent angiogenic cytokines, often alongside VEGF receptors (Fiedler et al., 1997; Padró et al., 2002; Kampen et al., 2013). Elevated VEGFA and VEGFC in particular have been linked to poorer prognosis (Kampen et al., 2013). AML cells thus establish a self-supporting “paracrine-autocrine” loop, involving autocrine VEGF secretion and activation of VEGFR2/3 to promote self-renewal, proliferation and survival (via distinct “internal” and “external” loop systems), plus concomitant paracrine promotion of angiogenesis on regional ECs. Other cells in the leukemic microenvironment, including MSCs and megakaryocytes, are meanwhile induced to express additional VEGF, further potentiating the positive feedback circuit (Pruneri et al., 1999; Dias et al., 2000; Casella et al., 2003; Kampen et al., 2013). Similarly, AML cells also express Angiopoietins (Ang-1, Ang-2) and their cognate receptor Tie2, again providing opportunity for autocrine propagation which appears operational in at least some AML cases (Wakabayashi et al., 2004; Reikvam et al., 2010).

Endothelial cells provide direct support to enhance leukemic proliferation, with evidence from transwell and direct contact experiments for AML cells providing reciprocal growth support to EC subtypes (Hatfield et al., 2009). Intriguingly, evidence has been presented for fusion of AML cells with ECs, in both mouse models and AML patients (Skinner et al., 2012; Cogle et al., 2014). Vascular-fused AML cells assumed EC-like characteristics, including up-regulation of CD105. Separately, AML cells could be induced to differentiate into *bona fide* EC-like cells *in vitro*, capable of recapitulating AML in xenotransplantation experiments (Cogle et al., 2014). These observations could contribute to the striking overlap in phenotype and function, and bidirectional crosstalk, between AML and ECs. Fusion could also form a reservoir for AML cells to evade chemotherapy and effect relapse.

Acute myeloid leukemia blasts have the ability to induce activation of the vascular endothelium through secretion of inflammatory cytokines, such as TNF-α and IL-1β. These induce expression of cell surface molecules on ECs, including ICAM-1, VCAM-1 and E-selectin, promoting adhesion of leukemic blasts to endothelium (Stucki et al., 2001). E-selectin is associated with the induction of proliferation and lineage commitment in HSCs (Winkler et al., 2012). Interestingly, upregulation of endothelial E-selectin in response to TNF-α released by AML blasts was recently shown to provide leukemic cells with a pro-survival signal through Akt/NF-κB signaling, conferring chemoresistance (Barbier et al., 2020). Additionally, activated endothelium increases proliferation and chemoresistance of AML cells through release of IL-8, presumably *via* Akt activation (Vijay et al., 2019).

#### Vascular Remodeling in AML

Acute myeloid leukemia progression has also been revealed to induce dramatic remodeling of BM vasculature. BM from AML patients displays significantly increased microvessel density, which is itself directly prognostic (Hussong et al., 2000; Padró et al., 2000; Kuzu et al., 2009). Interactions of AML blasts with ECs were reported to induce angiogenesis through VEGF and Notch/Dll4 signaling (Zhang et al., 2013), correlating with earlier observations of increased blood vessel formation in the BM of AML patients (Hussong et al., 2000). Using advanced intravital microscopy approaches two recent studies have demonstrated early, progressive, focal and spatially non-random patterns of vascular remodeling in murine models of human AML (Passaro et al., 2017; Duarte et al., 2018a). Both confirmed an overall expansion of the endothelial compartment and BM microvessel density specific to engraftment of AML in recipient mice. Passaro et al. (2017) showed this remodeling to differentially involve an increase in arteriolar (CD31^+^Sca1^*high*^) ECs, but loss of those corresponding to sinusoids (CD31^+^Sca1^*low*^). Remodeling was associated with disrupted sinusoidal structure, reduced overall vessel diameter, increased vascular permeability, and induction of poorly perfused, hypoxic regions; initially co-localizing with AML cells but becoming more pervasive throughout marrow with higher levels of leukemic marrow infiltration (Passaro et al., 2017).

**FIGURE 2:**
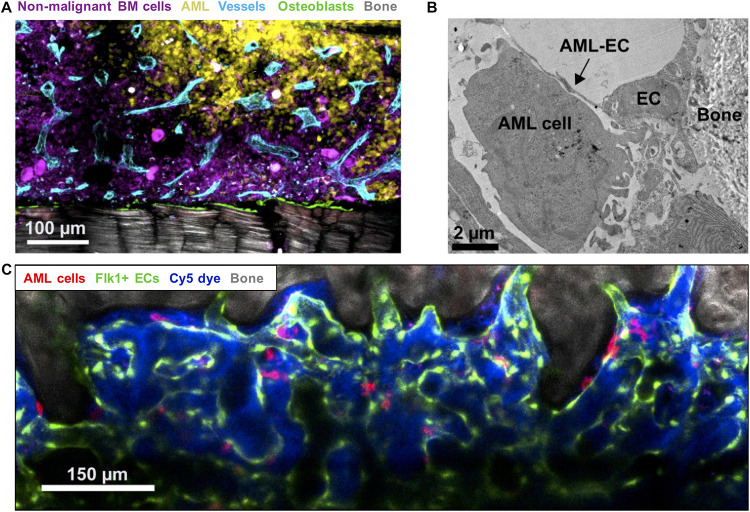
Imaging leukemic-microenvironment interactions. **(A)** Immunofluorescence of BM showing progressive infiltration by AML cells (YFP^+^ MLL-AF9^+^ AML; yellow) together with loss of non-malignant hematopoietic cells (mTomato^+^; purple) and remodeling of blood vessels (Endomucin^+^ vessels; cyan) and osteoblasts (Col2.3-CFP^+^ Ob; green) adjacent to bone (SHG; gray). **(B)** Transmission electron microscopy depicting AML-EC interactions in endosteal areas. **(C)** Combined confocal and multiphoton intravital microscopy of the calvarium BM showing surviving mTomato^+^ AML cells (red) surviving induction chemotherapy and interaction with Flk1^+^ ECs.

Duarte et al. (2018a) showed differential remodeling of endosteal (metaphyseal) versus central marrow (diaphyseal) endothelium: the former becoming progressively degraded in response to pro-inflammatory and anti-angiogenic factors released by endosteal AML cells, with the latter remaining highly vascularized throughout (Figure 2). While no distinction between different vessel subtypes was made, the reduction of sinusoidal ECs in AML reported by Baryawno et al. (2019) in their comprehensive stromal single-cell study implicates these as the likely subpopulation being disrupted. Perivascular and endosteal stroma became progressively abnormal and depleted with increasing blast burden, with formation and retraction of vessel sprouts evident in highly infiltrated areas leading ultimately to detachment of ECs and vascular collapse. Specific loss of endosteal vasculature was confirmed in BM from AML patients with > 80% blast involvement (Duarte et al., 2018a). Endosteal vascular remodeling is thus both dynamic and focal, directly accounting for two key disease features: loss of normal HSCs from endosteal areas no longer able to sustain them; and co-opting of these same spaces by leukemic cells, now able to survive/evade chemotherapy in a newly-transformed preferential niche.

Acute myeloid leukemia progression has additionally been associated with increased transendothelial cell migration that may facilitate cell ousting from the BM (Duarte et al., 2018a) and with an increase in vascular permeability (Passaro et al., 2017). The increased permeability is mainly driven by the stimulation of nitric oxide (NO) production in ECs, which in turn damages the BM endothelium (Passaro et al., 2017). Using both pharmacological and genetic approaches, Passaro et al. (2017) showed that inhibition of NO production in AML reduced vascular permeability and, correlating with the key role of the vascular endothelium supporting hematopoiesis and allowing drug delivery into BM, enhanced both HSC function and treatment response.

### Myelodysplastic Syndromes

Myelodysplastic syndromes refers to a heterogeneous group of clonal pre-leukemic myeloid HSC neoplasms characterized by increased BM cellularity but ineffective hematopoietic output, attributed to both aberrant differentiation and excessive apoptosis (Arber et al., 2016; Hellström-Lindberg et al., 2020). Clinical consequences are those of BM failure, with anemia, transfusion dependency, defective hemostasis and propensity to infection. Its natural history includes accrual of myeloblasts, with ∼30% of patients progressing to overt AML. Disease evolution is dependent on genetic, epigenetic and immunological factors (Albitar et al., 2002). Despite advances in understanding its molecular pathogenesis therapeutic options remain inadequate, for lack of effective means to actively manipulate and restore hematopoietic function. The only disease-modifying drug approvals in recent decades have been hypomethylating agents, which transiently improve HSC function and can delay AML transformation but are invariably not curative.

#### Mesenchymal Stem Cells in MDS

The concept of a diseased MDS BM niche is increasingly appreciated as fundamental to disease initiation and progression, with virtually all microenvironmental components having been implicated *via* diverse mechanisms. Several studies have identified presence of cytogenic abnormalities, impaired capacity to support hematopoiesis and dysfunctional generation of cytokines essential for maintaining hematopoiesis, including LIF and Angptl4, in cultured MSCs *ex vivo* (Blau et al., 2011; Zhao et al., 2012; Medyouf et al., 2014). MDS MSCs were shown to preferentially support CD34^+^ engraftment with a myeloid bias when co-transplanted in murine models, suggesting a supportive role in MDS pathogenesis (Medyouf et al., 2014). MSC function is also influenced by BM iron overload, a state associated with ROS-mediated oxidative stress and that characterizes MDS independent of transfusional siderosis (Moukalled et al., 2018). Iron recycling, critical for erythropoiesis and to prevent oxidative stress, is undertaken by the action of heme oxygenase-1 on heme almost exclusively within perivascular BM macrophages (Korolnek and Hamza, 2015), which in MDS are dysfunctional and expanded in line with BM iron levels (Nybakken and Gratzinger, 2016). These have extensive contact with CD271^+^ MSCs and communicate *via* exosome-mediated transfer of microRNAs and mitochondria (Phinney et al., 2015), with a direct role in regulating the trilineage differentiation potential of MSCs (Vanella et al., 2010). Accordingly, BM MSCs from iron overloaded MDS patients displayed a range of abnormalities, including increased apoptosis, mitochondrial fragmentation and enhanced autophagy; these were ROS-dependent, and apparently mediated by activation of AMP-activated protein kinase (AMPK) (Zhang et al., 2015; Zheng et al., 2018). The MSC dysfunction was associated with defective support of co-cultured hematopoietic cells (Zhang et al., 2015). More broadly, exacerbated BM oxidative stress could further promote genomic instability of the MDS hematopoietic cells and clonal evolution toward AML. Transgenic mouse experiments have also shown that mutations in niche cells, including deficiency of Dicer1 in osteoprogenitors and activation of β-catenin in mature osteoblasts, could induce an MDS-like phenotype (Raaijmakers et al., 2010). Defective adhesion of myeloid progenitors to BM extracellular matrix has also been implicated. Matrix metalloproteinases are proteolytic enzymes that degrade extracellular substrates (including collagens, fibronectin and laminin), but also process numerous inflammatory chemokines and cytokines. Several, most notably MMP2 and MMP9, have been widely (albeit variably) reported as dysregulated in MDS, with consequences including disruption of integrin-mediated adhesion/signaling and altered generation of death proteins (e.g., TNF-α, Fas-ligand) (Iwata et al., 2007; Travaglino et al., 2008; Chaudhary et al., 2016; Youn et al., 2019). Intriguingly, MMP9 inhibition restored erythropoiesis in models of del(5q) MDS, through suppression of TGF-β signaling (Youn et al., 2019).

#### Endothelial Cells in MDS

A specific role for vascular niche components has been harder to establish, partly due to inconsistent EC subtype definitions and lack of clarity on phenotypic verification of endothelial progenitors (Huizer et al., 2017). However, a growing body of evidence supports the existence of a dysfunctional vascular niche, and its potential to promote MDS initiation, progression and therapeutic resistance. Alteration of the vascular niche is readily appreciated in the morphological feature termed “abnormal localization of immature precursors” (ALIP), a histopathological hallmark of MDS and itself a useful diagnostic feature (Mangi et al., 1991). This refers to the observation of myeloid HSPCs abnormally localized in the BM interstitium, rather than paratrabecular endosteal niche as typical in healthy BM. In the context of myelofibrosis ALIP disorganization can promote release of immature precursors into circulation, representing a direct mechanism by which a faulty vascular niche contributes to disease progression (Lataillade et al., 2008; Bousse-Kerdilès, 2012). A similar phenotype has been described in AML, in which loss of HSCs specifically from endosteal niches and their abnormal release into the periphery, as well as protection of malignant clones from chemotherapy, have been described (Passaro et al., 2017; Duarte et al., 2018a).

Analogous to AML, MDS patients display increased BM microvascular density (Pruneri et al., 1999; Korkolopoulou et al., 2001), which correlated variously with higher BM myeloblast percentage (Pruneri et al., 1999), more advanced disease subtypes (Pruneri et al., 1999; Alexandrakis et al., 2005; Wimazal et al., 2006; Kim et al., 2016) and higher prognostic risk group (Savic et al., 2012), implicating an association with progression toward leukemia. This might be explained by secretion of angiogenic growth factors from clonal MDS cells, with higher serum concentrations of VEGF, Ang-1, angiogenin, β-FGF, HGF, TNF-α, EGF, and IL-6, amongst others, reported in MDS patients (Brunner et al., 2002; Alexandrakis et al., 2004, 2005; Feng et al., 2011; Pardanani et al., 2012).

Of these, the VEGF family’s role has been best characterized. VEGFA is overexpressed and secreted by MDS myelomonocytic precursors, alongside its receptors VEGFR1 (FLT-1) and VEGFR2 (KDR) (Dias et al., 2000; Casella et al., 2003). Co-expression has been observed within ALIPs in MDS biopsy specimens, as also in monocytic precursors in CMML and myeloblasts in AML (Casella et al., 2003). The ratio of myeloblasts to monocyte precursors in BM also correlates with VEGF expression, thereby coupling more advanced disease status with higher angiogenic potential (List et al., 2004). Similarly, serum levels of VEGF and also Ang-1, were higher in patients with more advanced MDS and CMML, at levels comparable to AML (Brunner et al., 2002). As in AML, VEGF dysregulation in MDS promotes both paracrine signaling to mediate regional microenvironmental remodeling, and autocrine growth and proliferation of MDS progenitors (Albitar, 2001; Bellamy et al., 2001). Indeed, VEGF stimulation enhanced *ex vivo* leukemic colony formation of primary CMML and advanced-stage MDS samples, effects reversed upon treatment with a VEGF-neutralizing antibody (Bellamy et al., 2001). Beyond promoting vascular remodeling, paracrine effects of MDS-derived VEGF on ECs also promote their secretion of supportive myeloid growth factors, for example GM-CSF (Fiedler et al., 1997), contributing further to survival and progression of the MDS clone. Finally, VEGF induces vascular permeability in sinusoidal ECs, which promotes HSPC cycling, migration and differentiation at the expense of their fitness and function, with increased apoptosis and myeloid output bias observed in murine transplantation models (Itkin et al., 2016). Both are prominent features of MDS hematopoiesis, highlighting another potential mechanism by which excessive VEGF could contribute to MDS pathobiology.

Endothelial cells in MDS exhibit genetic, transcriptional and epigenetic dysregulation, sometimes resembling abnormalities found in the clonal MDS hematopoietic cells. In a study of stringently sorted circulating ECs (CD34^+^/CD45^–^/CD146^+^) from MDS patients, FISH confirmed 39–84% of cells to carry identical cytogenetic abnormalities to those characterizing the respective MDS hematopoietic clones (Porta et al., 2008). Whether this reflected a common derivation, reprogramming of clonal hematopoietic cells by angiogenic factors, or cell-fusion events, was not explored. Another study described putative MDS-related transcriptional and methylation signatures for endothelial colony forming cells (ECFCs) isolated from low-risk MDS patients (Teofili et al., 2015). These cells, present at substantially higher numbers in MDS patients than controls, exhibited a DNA promoter CpG hypermethylation phenotype across four genes frequently hypermethylated in MDS blood cells (*CDKN2B, DAPK1, CDH1*, and *SOCS1*); none were methylated in ECFCs from any of 14 healthy individuals. Microarray revealed prominent up-regulation of genes encoding adhesion molecules (ICAM-1, VCAM-1, L-Selectin, and VWF), the nitric oxide generator NOS3, TNFSF10 (which promotes apoptosis), and BMP (a secreted ligand of TGF-β and powerful inducer of erythroid differentiation). Notable down-regulated genes included apoptotic regulators (*BCL2L1* and *CASP2*), the cell cycle driver *CCND1* and multiple components of the Wnt signaling pathway. Since recombinant WNT3A partially restored the ECs’ ability to support hematopoiesis, ineffective Wnt signaling might mediate the aberrant attachment of hematopoietic cells to the vascular niche and the consequent impacts on HSC function. Interestingly, normal CD34^+^ cord blood HSPCs co-cultured with MDS-derived (versus healthy control) ECFCs displayed markedly reduced proliferation and multilineage differentiation potential, associated with aberrant expression of key genes associated with granulomonocytic and erythroid differentiation, including *SPI1, MPO, TFR*, and *GPIb* (Teofili et al., 2015). In another study, MDS MSCs displayed reduced expression of fundamental HSC maintenance genes, including *CXCL12* and *VEGFA*, whereas by contrast MDS ECs up-regulated *CXCL12, SCF*, and *LIF* (Abe-Suzuki et al., 2014). This suggested that MSCs lost HSC supportive capacity, but with compensation by the endothelial compartment, indicating a dynamic redistribution of supportive roles within the dysfunctional MDS niche.

## Therapeutic Targeting of the Vascular Niche

**FIGURE 3:**
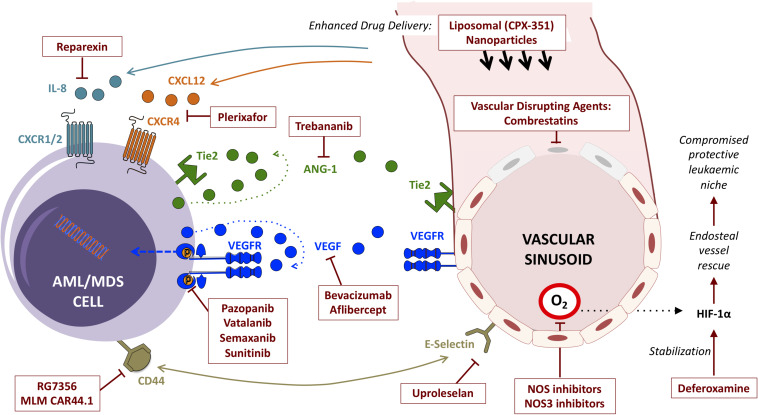
Summary of strategies (and exemplar agents) for the therapeutic targeting of interactions between myeloid malignancies and BM vascular niche components.

Despite being generally chemosensitive diseases, clinical outcomes for patients with AML, MDS and other myeloid malignancies remain suboptimal. Deep molecular insights have added a host of epigenetic, immunotherapy and other targeted agents to conventional chemotherapy in the treatment armamentarium, with a run of recent FDA approvals (DiNardo et al., 2018; Fiorentini et al., 2020). Most of these directly target cell-intrinsic vulnerabilities in cells of the malignant clone itself, yet this new wealth of treatment options has yet to translate into a meaningful improvement in overall outcomes. Disease relapse remains common, and cure without allogeneic stem cell transplantation remains elusive for most in this context. Emerging appreciation of the critical role played by the aberrant leukemic microenvironment in sustaining leukemic cell survival has opened another front, highlighting new potential targets for adjunctive therapeutics. Prominent amongst these are strategies targeting components of the vascular niche and their bidirectional interactions with malignant myeloid cells (Figure 3).

### Targeting the VEGF Axis

By far the most studied are the “classical” anti-angiogenic drugs, particularly those targeting the binding of VEGF to its receptors. Some of these have realized their potential and been approved across a range of solid cancers (Vasudev and Reynolds, 2014). Several observations suggested a potential role in treating myeloid malignancy. The hypervascular AML BM microenvironment is characterized by increased BM microvessel density, directly mediated by the AML cells themselves, which correlates with prognosis and can revert to normal at remission (Padró et al., 2000; Kuzu et al., 2009; Weidenaar et al., 2011). Moreover, AML cells frequently secrete angiogenic cytokines, and express their cognate receptors (Fiedler et al., 1997; Dias et al., 2000; Padró et al., 2002; Loges et al., 2005; Hou et al., 2008; Weidenaar et al., 2011). They also express adhesion molecules mediating physical interaction with ECs (e.g., VLA-4/VCAM-1; CD44/E-selectin), and blasts located in close proximity to ECs are conferred with relative chemoresistance (Hazlehurst and Dalton, 2001; Jacamo et al., 2014). Potential for broad applicability across disease indications and subtypes is another attractive feature.

Despite theoretical promise, however, results from clinical trials with anti-angiogenic agents in myeloid malignancies have been disappointing. Bevacizumab is a monoclonal antibody targeting VEGF that is licensed for a range of solid tumors. Following preclinical confirmation of decreased proliferation of AML blasts *ex vivo* (Karp et al., 2004; Zahiragic et al., 2007), early trials as monotherapy in AML demonstrated capacity to reduce VEGF expression within BM, but without impacting blast burden or outcome (Zahiragic et al., 2007). A phase 2 trial in 171 patients found no advantage for addition of bevacizumab to standard 7 + 3 daunorubicin plus cytarabine induction chemotherapy (Ossenkoppele et al., 2012). However, another study of 48 patients in the relapsed/refractory setting yielded a better-than-expected complete remission (CR) rate of 33% using a time-sequential strategy of cytarabine and mitoxantrone, followed by bevacizumab (Karp et al., 2004). This might indicate a critical timing or sequencing dependency for optimal deployment of anti-angiogenic therapies as adjuncts to conventional cytotoxics. Further research is required to better understand and optimally exploit this.

Other agents targeting VEGF have been similarly disappointing. Aflibercept is a high-affinity chimeric soluble decoy receptor, with the extracellular immunoglobulin-like domains of VEGFR-1 and -2 fused to the human Ig Fc segment. It thus acts as a “VEGF trap,” with 10-fold higher affinity for VEGFs than bevacizumab and activity against all isoforms (Holash et al., 2002). Aflibercept reduced AML progression in human AML murine xenograft models (Lal et al., 2010), but a phase 2 clinical trial was prematurely halted due to lack of efficacy, with no hematological responses observed (Kirschbaum et al., 2018). Of note, the study population, comprising MDS and MDS/MPN “Overlap” syndromes following hypomethylating agent failure, is notoriously difficult to treat, with dismal prognosis and low response rates across investigational studies. Aflibercept has not yet been evaluated in AML or earlier stage MDS patients.

Whereas bevacizumab and aflibercept prevent binding of VEGF-A to its receptors, an alternative strategy is to target the downstream intracellular signaling cascades activated upon ligand binding. Several tyrosine kinase inhibitors (TKIs) count among their targets receptor TKs involved in conveyance of angiogenic signaling.

Pazopanib is an orally bioavailable TKI of VEGFR (−1, −2, and −3), PDGFR-α, PDGFR-β, and KIT (Hamberg et al., 2010). It is approved for renal carcinoma and soft tissue sarcoma, in which it impairs perfusion of the solid tumor. Pazopanib’s kinase selectivity profile suggests multiple concurrent potential anti-leukemic mechanisms, given the dominant expression of KIT (CD117) on blasts from ∼60–70% of AMLs, and expression of PDGFR-α and –β in 45% and > 90% of AMLs, respectively, in addition to any VEGFR-targeting anti-angiogenic effect (Ikeda et al., 1991; Foss et al., 2001). Having observed low nanomolar *in vitro* cytotoxicity in AML cell lines, the German phase 2 PazoAML trial reported modest efficacy as monotherapy in a relapsed/refractory AML cohort, including two partial remissions (PR) with > 50% blast reduction (Kessler et al., 2019). Although disappointing, these outcome data are comparable to standard-of-care therapies (e.g., low dose cytarabine) in this setting, hinting at a possible efficacy signal in at least a subset of AMLs. Interestingly, BM microvessel density was not significantly reduced after 28 days treatment and showed no correlation with clinical response, implying activity through mechanisms unrelated to VEGFR inhibition. Pazopanib is included amongst >50 therapeutic options in a drug sensitivity assay and genomics-guided personalized therapy trial for relapsed/refractory AML currently enrolling (NCT02551718). This might illuminate a group of patients for whom it could hold particular promise.

Vatalanib similarly targets multiple VEGFRs and PDGFRs, with relatively greater selectivity against VEGFR-2 and *ex vivo* activity against CD34^+^ AML blasts, *via* inhibition of the PI3K/Akt pathway (Weidenaar et al., 2013). Whilst it has shown promise in metastatic solid cancers, and a 29% CR rate in combination with standard induction chemotherapy in a phase 1 AML trial (Roboz et al., 2006), a phase 2 CALBG Alliance study in MDS reported minor hematological improvement in only 5% of patients, with considerable toxicity (Gupta et al., 2013), and vatalanib has not been actively pursued in myeloid malignancy.

Semaxanib (SU5416) is another TKI that targets VEGFRs and KIT (Fong et al., 1999; Smolich et al., 2001), but with additional activity against FLT3: a cytokine receptor mutated in ∼30% of AML patients, resulting in constitutive signaling activation and a highly proliferative, poor prognosis leukemia (Yee et al., 2002). Semaxanib’s TK profile makes it an attractive candidate for AML, with potential for multiple concurrent anti-leukemic effects. Semaxanib also demonstrated anti-proliferative effect *in vitro via* inhibition of both VEGFR2 and Akt signaling (List et al., 2004). Again, however, early clinical translation has been disappointing. A phase 2 study in 43 relapsed/refractory c-KIT^+^ AML patients yielded one transient CR (of 2 months duration) and seven PRs (all lasting < 5 months) (Fiedler et al., 2003). Encouragingly, higher VEGF expression was associated with response and also with BM microvessel density reduction, implying a therapeutic on-target effect. Responses were seen in 6/16 (38%) of VEGFR2^+^AML patients, as compared with 0/7 VEGFR2^–^ cases, suggesting a potential biomarker for precision targeting. Notably, responses were not observed in any FLT3-mutant cases, suggesting against clinically relevant FLT3-directed activity. Unfortunately, due to discouraging results across potential indications the development program was discontinued.

A related TKI, sunitinib (SU112348), inhibits VEGFR1/2 and was developed as an anti-angiogenic drug, yet displays broad activity also against KIT, PDGFRα/β, CSF1R, LCK, and FLT3 (Roskoski, 2007), with potent activity in AML models both *in vitro* and *in vivo* (O’Farrell et al., 2003). It is now licensed for renal carcinoma and some gastrointestinal stromal and pancreatic neuroendocrine tumors. Sunitinib showed promise in early phase AML trials, especially in a FLT3-mutant context. Responses were seen in all of four FLT3-mutant relapsed/refractory AML patients in a small phase 1 (Fiedler et al., 2005), and a phase 1/2 elderly first-line combination study, combined with standard chemotherapy followed by 2 years sunitinib maintenance, reported historically favorable CR rate (59%) and survival outcomes (median overall survival 1.6 years) (Fiedler et al., 2015). Exploratory studies focused largely on dephosphorylation of FLT3, KIT and Akt; the relative contribution of VEGFR blockade was not evaluated. Dose-limiting cardiovascular toxicities are recognized for sunitinib across indications, although encouragingly no grade 3/4 cardiac events were reported in the combination study, despite concomitant anthracycline exposure in an elderly population (Fiedler et al., 2015). Clinical trials are ongoing.

### Targeting Angiopoietins

Alongside the VEGF family, the Angiopoietins (Ang-1, Ang-2) and their shared receptor (Tie2/TEK) are the most specific inducers of angiogenesis dysregulated in myeloid leukemia (Fagiani and Christofori, 2013). Tie2 is frequently overexpressed in AML, including on leukemic blasts themselves, whilst both Ang-1 and Ang-2 are overexpressed in BM and patient serum in some AML patients (Müller et al., 2002; Watarai et al., 2002; Schliemann et al., 2006, 2007). High expression of Ang-2 was a strong independent prognostic factor in at least three AML studies (Loges et al., 2005; Schliemann et al., 2006, 2007). As with VEGF and its receptor, at least some primary AML blasts express both Angiopoietin and Tie2, and establish a dependent autocrine stimulation loop (Reikvam et al., 2010; Trujillo et al., 2012). Overall, the role of the Ang1/Ang2/Tie2 axis in AML appears somewhat complex, likely involving reversal of the balance of Ang1 versus Ang2 (which have distinct and possibly opposing agonist/antagonist functions) as they compete for binding with Tie2, and is probably further influenced by prevailing VEGF-A levels (Loges et al., 2005). Blocking Angiopoietins from binding Tie2 decreased proliferation of AML blasts *ex vivo* (Reikvam et al., 2010), highlighting a promising therapeutic strategy. Trebananib (AMG-386) is a neutralizing peptibody that sequesters Ang-1 and Ang-2, preventing their interaction with Tie2. It has been trialed in a range of mostly solid cancers, but has shown promise in preclinical AML models. A phase 1b trial of trebananib with or without cytarabine reported tolerable safety profile and lack of neutralizing antibody formation, but only a single PR among 24 AML patients (Wang E.S. et al., 2014). No phase 2 or novel combination trials in myeloid malignancy have been registered.

Overall, efficacy of classical anti-angiogenic agents as monotherapy in established AML appears disappointingly limited, despite successes in other areas of oncology. Combination strategies with cytotoxic or other targeted agents, potentially in a personalized fashion, must be sought if they are to have any meaningful role in the future treatment landscape of myeloid malignancies.

### Vascular Disrupting Agents

As an alternative to preventing angiogenesis, another class of compounds directly target and break down pre-existing ECs, disrupting vascular architecture and impairing blood flow to tumor cells and their microenvironment.

The combretastatins are naturally occurring microtubule-destabilizing agents, structurally similar to colchicine, derived from bark of South African *Bush Willow*. They bind to the β-tubulin subunits in proliferating ECs, causing disruption of mitochondrial membrane potential, release of pro-apoptotic mitochondrial membrane proteins and DNA fragmentation, resulting in EC death through a caspase-dependent mechanism (Petit et al., 2008). The first-in-class, CA4P, is efficacious in animal models of human solid cancers, inducing vascular collapse and restricting perfusion of tumor cells. Preclinical results were encouraging in a murine model of AML engrafted with several cell lines, in which CA4P inhibited proliferation and circulation of leukemic cells and attenuated perivascular leukemic infiltration, significantly prolonging survival (Petit et al., 2008).

Another combretastatin, CA1P (OXi4503), displays more potent vascular disruption and antitumor activity, with additional cytotoxic effects purportedly through formation of reactive ortho-quinones (Folkes et al., 2007). In xenograft models of human myeloid sarcoma (a solid tumor variant of AML) OXi4503 successfully disrupted tumor vasculature, but with a residual (and richly vascular) circumferential leukemic rim. Addition of bevacizumab prevented reactive compensatory rim formation, suggesting a possible synergy of anti-angiogenic and vascular disruptive agents, at least in the context of solid tumor AML (Madlambayan et al., 2010). The combination was effective too at preventing engraftment in a murine model of systemic AML, demonstrably due to a multitargeted mechanism combining microvessel density reduction and direct cytotoxicity mediated by ROS-induced apoptosis. Phase 1B results were recently reported for OXi4503 in combination with cytarabine in 29 AML and MDS patients with relapsed/refractory disease. Safety profile was encouraging, with a 19% overall response rate observed, including four complete morphological remissions (Uckun et al., 2019). Phase 2 evaluation is ongoing (NCT02576301).

Many other microtubule colchicine binding site agents have been identified that also show potent vascular disrupting and anti-neoplastic activity. These include Dolastatin 10 (derived from a marine mollusc), its synthetic derivative TZT-1027, and the prodrug ZD6126: although the latter’s development program was halted due to unacceptable cardiotoxicity in early phase human trials (Ji et al., 2015; Cogle et al., 2016).

### Disrupting Adhesion to Endothelial Cells (and Its Consequences)

Ultimately the main value of targeting the vascular niche might prove to be in physically dislodging leukemia cells from their protective niche, thereby sensitizing to cytotoxic or other leukemia cell-directed therapies. Indeed, by comparison with anti-angiogenic agents, strategies targeting the anchoring of leukemia to stromal cells, in particular the CXCR4-CXCL12 axis, have been relatively more successful (Cho et al., 2017). Plerixafor (AMD3100), a small molecule inhibitor of CXCR4, has been in wide usage for peripheral blood stem cell mobilization for many years, and shown efficacy in numerous combination trials in AML (Maganti et al., 2020). Several other agents blocking the interaction of CXCR4 with its ligand CXCL12 (e.g., AMD3465, BL-8040, LY2510924, CTCE-9908, and ulocuplumab) are also in early phase trials (Cho et al., 2017). Similarly, drugs targeting the interaction between VCAM-1 (on MSCs) and VLA-4 (on AML cells) have undergone preclinical evaluation. The humanized VLA-4 monoclonal antibody natalizumab (Tysabri), extensively used in autoimmune diseases, showed high efficacy in AML xenograft models (Hsieh et al., 2013) although concerns over JC virus-associated progressive multifocal leukoencephalopathy limited its application in the AML setting (Bloomgren et al., 2012). AS101, a non-toxic tellurium compound which induces redox inhibition of VLA-4 upon binding to fibronectin, also restored chemosensitivity and prolonged survival in AML xenografts (Layani-Bazar et al., 2014).

The most promising analogous candidate in the vascular niche is E-selectin (CD62E). It has important roles in maintaining normal HSC homeostasis (Winkler et al., 2012), but was recently also shown to also trigger survival signaling through the Akt and NF-κB pathways to promote and sustain AML cells (Barbier et al., 2020). Inflammatory mediators released by AML cells, including TNF-α, directly up-regulate E-selectin expression, high levels of which were associated with dramatically (12-fold) higher resistance to chemotherapy *in vivo*. Therapeutic inhibition with the E-selectin inhibitor uproleselan (GMI-1687) has shown impressive preclinical efficacy, synergizing with cytarabine to double survival duration in AML mouse models (Barbier et al., 2020), and subsequently reported encouraging results including an impressive CR rate of 41% in a phase 1/2 clinical trial of relapsed/refractory AML (DeAngelo et al., 2018). International randomized, placebo-controlled phase 3 trials in combination with standard chemotherapy in first-line (NCT03701308) and relapsed/refractory (NCT03616470) settings are ongoing. Meanwhile, uproleselan has been granted Breakthrough Therapy and Fast Track designations from the FDA, underscoring its strong promise in AML. Its potential may extend to other myeloid malignancies: for example, synergy was demonstrated with imatinib to extend survival in preclinical models of chronic myeloid leukemia (CML), *via* decreased contact time of leukemia cells with BM endothelium (Godavarthy et al., 2019).

CD44, a major ligand of E-selectin, is itself a promising candidate target. This extensively glycosylated adhesion molecule is widely expressed in hematopoiesis, most abundantly on HSCs, and is also up-regulated on AML leukemia stem cells (LSCs). Jin et al. (2006) demonstrated selective eradication of LSCs in murine xenotransplantation models of human AML using the activating CD44-targeted monoclonal antibody H90, interfering with AML cell homing and directly altering LSC fate. A phase 1 trial of the humanized monoclonal antibody RG7356 showed it to be safe (maximum tolerated dose not reached) with modest efficacy (Vey et al., 2016). Although direct CD44-targeting immunotherapy has not advanced further, the isoform variant 6 of this heavily glycosylated adhesion molecule has emerged as an attractive target for chimeric antigen receptor (CAR) T cell-based therapies. Expressed on AML but notably absent on normal HSCs, CAR-Ts designed against CD44v6 have demonstrated preclinical promise (Casucci et al., 2013), with the autologous product MLM-CAR44.1 currently in a phase 1/2a trial in AML and multiple myeloma [(Carrabba et al., 2018); NCT04097301].

IL-8 is one of the most highly secreted factors in response to AML cell-mediated EC contact activation and has been shown itself to support AML cell proliferation and chemoresistance through a paracrine positive feedback mechanism. A small molecule screen identified a lead candidate IL-8 inhibitor, NCI34255, that abrogated AML cell proliferation and restored cytarabine sensitivity *in vitro via* a mechanism involving Akt pathway inhibition (Vijay et al., 2019). These results have yet to be demonstrated *in vivo*. However, an inhibitor of the IL-8 receptors CXCR1/2, Reparixin, is undergoing clinical trials having shown early phase promise in breast cancer (Ruffini, 2019). Thus targeting the IL-8/CXCR1/CXCR2 axis might be another promising avenue for clinical exploration in AML, as well as other myeloid malignancies in which high IL-8 expression has been pathologically implicated, including MDS (Shi et al., 2019), CMML (Niyongere et al., 2018), and myelofibrosis (Tefferi et al., 2011).

### Restoration of the Compromised Endosteal Niche

The broadly disappointing results targeting angiogenic factors and individual vascular niche components underlie the more complex, nuanced relationship with angiogenesis for leukemias than for solid cancers. In the latter, survival of the growing tumor relies upon coordinated neovascularization to maintain adequate perfusion. By contrast, recent studies have confirmed that AML cells actively remodel the vasculature to create and directly co-opt regions of defective perfusion and hypoxia, whilst simultaneously rendering these regions hostile to residual HSCs, thereby supporting leukemic cells to outcompete residual normal hematopoiesis. Critically, endosteal vessel changes and generation of protective hypoxic niches appear to be primary events in AML pathogenesis that, once established, are not reversible with conventional therapy (Passaro et al., 2017; Duarte et al., 2018a; Figure 2). Leukemic mice displayed higher levels of VEGF-A associated with observations of sprouting angiogenesis, although these sprouts did not progress to form new blood vessels (Duarte et al., 2018a). Taken together, alongside recognition that AML cells release a diverse array of both pro- and anti-angiogenic factors, these observations might explain the relative resistance of established AML to conventional anti-angiogenic therapy.

Fundamental insights into the temporal and spatial nature of AML-induced vascular remodeling (Passaro et al., 2017; Duarte et al., 2018a) have raised the intriguing prospect of directly manipulating this process to potentially restore the compromised vascular niche to one more hostile to leukemia cells and more hospitable to normal hematopoiesis. One approach could exploit the ability of deferoxamine, a prolyl-4-hydroxylase inhibitor, already used as an iron chelator for transfusional siderosis (including in some patients with myeloid malignancies), to induce endosteal vessel expansion *via* stabilization of HIF-1α (Kusumbe et al., 2014, 2016). The concept was tested in murine AML models, in which deferoxamine treatment of mice with established leukemia successfully enhanced endosteal vessel formation, with substantially higher numbers of HSCs within trabecular-rich metaphyses (but not diaphysis) due at least in part to enhanced HSC homing ability (Duarte et al., 2018a). Although mice with fully established leukemia showed no difference in AML blast infiltration or survival after deferoxamine therapy, a parallel genetic manipulation approach to rescue endosteal vasculature in another mouse model, through inducible activation of Notch signaling in ECs before administration of doxorubicin and cytarabine, markedly re-sensitized AML cells to induction chemotherapy (Duarte et al., 2018a). A similar sequential strategy, using deferoxamine or other Notch-activating agents, could theoretically restore blood supply to compromised protective niches and thus hold great promise as adjunctive therapy for treating established AML in patients. If validated, this would represent a conceptual paradigm shift in AML induction practice.

Hypoxic niches generated by the vascular remodeling process are likely fundamental to supporting leukemic progression and evasion from chemotherapy, but could present other opportunities for therapeutic modulation. Among the genes most highly up-regulated in endothelial cells following human AML engraftment in a murine model was *Nox4*: a NADPH oxidase involved in response to hypoxia *via* production of ROS, activation of NOS3 and release of NO (Passaro et al., 2017). Neither Nox4 overexpression, nor pervading high levels of ROS or NO within BM, were reversed by conventional chemotherapy in this model, and persisting high NO levels in post-treatment BM samples from primary AML patients was strongly predictive for treatment failure. In two murine models of MLL-rearranged AML, leukemia initiation was significantly delayed and disease expression ameliorated in *Nos3*^*ko*^ recipient mice (Passaro et al., 2017). Loss of *Nos3* cooperated with cytarabine to impair AML engraftment and correct endothelial abnormalities. NOS inhibitors inhibited leukemic progression in a human AML (ML1 cell line) xenotransplantation model, but only when combined with cytarabine. The effect was, however, significantly greater than that of chemotherapy alone, and also restored vascular function, corrected aberrant hypoxic patterns, and supported normal HSC function and repopulation ability (Passaro et al., 2017). Thus, targeting of hypoxia through, for example inhibitors of NOS (already in clinical trials) or specifically of NOS3 (in development) could represent a valuable strategy to enhance the effect of conventional chemotherapy.

### Improved Drug Delivery to the Protective Malignant Niche

A potential alternative to releasing leukemic cells from their protective niche is to exploit the rapid advances in biotechnology to more effectively deliver existing drugs to these regions. Liposomal formulations can deliver more robust and consistent doses to BM niches, the most prominent example being CPX-351, a liposomal formulation combining daunorubicin and cytarabine that is now standard-of-care first-line therapy for some AML subgroups (Tzogani et al., 2020). Nanoparticles as a delivery system to sequestered sites holds great promise (Vinhas et al., 2017), although remains largely experimental and few have yet reached clinical trials. There is a growing body of data suggesting that these can enhance drug delivery to malignant cells within protective niches. For example, a recent report described successful targeted delivery of arsenic trioxide encapsulated in novel dual oligopeptide-conjugated nanoparticles, to effectively eliminate primitive CML stem cells within endosteal and vascular niches in an *in vitro* model (Fan et al., 2020).

A promising approach for enhanced drug delivery is the direct targeting of hypoxic BM niches through use of hypoxia activated prodrugs (HAPs). Extravascular BM pO_2_ is reported to be in the range of 10–25 mmHg (mean ∼17 mmHg, about 2% O_2_) irrespective of the debate surrounding localization of HSC to areas of more profound hypoxia (Christodoulou et al., 2020). HAPs are inactive until metabolized by oxygen-inhibited enzymes (typically 1–2 electron oxidoreductases) to produce cytotoxic (usually DNA reactive) agents. The HAP evofosfamide (TH-302) has demonstrated drug-dependent cytotoxicity against primary AML cells and the leukemia stem-cell enriched compartment (CD34^+^ CD123^+^ cells) under hypoxic conditions with minimal cytotoxicity against normal BM samples. *In vivo* utility was confirmed in NOD/Scid/IL2RγKO (NSG) mice transplanted with primary AML cells (Benito et al., 2016). In Ph1/2 clinical studies the HAP PR104 demonstrated anti-leukemic activity in AML patients, with the main reported side effect being myelosuppression. Hypoxia was elegantly demonstrated in a concurrent biomarker study, in which high expression of the hypoxia tracer pimonidazole was found in the leukemic cells within BM biopsies of AML patients (Benito et al., 2016).

## Conclusion and Future Directions

The BM vascular niche represents a dynamic interface with emerging hematopoiesis, with extensive crosstalk in both health and disease. A relative complexity for the bidirectional interplay has clearly emerged from a series of transformative studies, supported by advances in modeling and intra-vital imaging techniques, amongst others. These have revealed key insights into mechanisms of leukemogenesis, and highlighted a host of candidate approaches to modulate critical dependencies to therapeutic effect. Rapid advances in single cell genomic, transcriptomic and epigenomic technologies now enable multi-omic evaluation of highly-refined subsets of distinct microenvironmental components, prospectively and concurrently, and in response to specific perturbations. Along with the discovery potential for modern gene editing technologies, a new wave of insights into microenvironmental influences of hematopoiesis are anticipated.

Whilst early trials with anti-angiogenic and vascular disrupting agents were disappointing there are hints of meaningful efficacy, particularly for a priming role adjunctive to cytotoxic chemotherapy in at least some patients. Appreciation of biomarkers predictive for response could prospectively identify patients most likely to benefit from vascular-targeting therapies. Rational combinations targeting multiple factors, perhaps in deliberate sequence, could re-invigorate optimism for a role in the future treatment arsenal. Newer, more sophisticated approaches to directly modulate the malignant vascular niche may hold greater promise still. Caution must, however, be exercised for unintended consequences of modulating the AML microvasculature. Predictable cardiovascular side effects have been observed and, indeed, been responsible for the closure of some development programs. Determining how these risks manifest in AML patients, who may have other particular risk factors (e.g., anthracycline exposure; *TET2*-mutated clonal hematopoiesis), will mandate design of specific endpoints and exploratory research in future trials.

## Author Contributions

LM, JS, KB, DD, ES, and DW together all conceived, wrote, and revised the manuscript. All authors contributed to the article and approved the submitted version.

## Conflict of Interest

The authors declare that the research was conducted in the absence of any commercial or financial relationships that could be construed as a potential conflict of interest.

## References

[B1] Abegunde S. O. Buckstein R. Wells R. A. Rauh M. J. (2018). An inflammatory environment containing TNFα favors Tet2-mutant clonal hematopoiesis. *Exp. Hematol.* 59 60–65. 10.1016/j.exphem.2017.11.002 29195897

[B2] Abe-Suzuki S. Kurata M. Abe S. Onishi I. Kirimura S. Nashimoto M. (2014). CXCL12+ stromal cells as bone marrow niche for CD34^+^ hematopoietic cells and their association with disease progression in myelodysplastic syndromes. *Lab. Invest.* 94 1212–1223. 10.1038/labinvest.2014.110 25199050

[B3] Acar M. Kocherlakota K. S. Murphy M. M. Peyer J. G. Oguro H. Inra C. N. (2015). Deep imaging of bone marrow shows non-dividing stem cells are mainly perisinusoidal. *Nature* 526 126–130. 10.1038/nature15250 26416744 PMC4850557

[B4] Albitar M. (2001). Angiogenesis in acute myeloid leukemia and myelodysplastic syndrome. *Acta Haematol.* 106 170–176. 10.1159/000046613 11815714

[B5] Albitar M. Manshouri T. Shen Y. Liu D. Beran M. Kantarjian H. M. (2002). Myelodysplastic syndrome is not merely “preleukemia”. *Blood* 100 791–798. 10.1182/blood.v100.3.791 12130488

[B6] Alexandrakis M. G. Passam F. H. Pappa C. A. Damilakis J. Tsirakis G. Kandidaki E. (2004). Serum evaluation of angiogenic cytokines basic fibroblast growth factor, hepatocyte growth factor and TNF-ALPHA in patients with myelodysplastic syndromes: correlation with bone marrow microvascular density. *Int. J. Immunopath. Pharmocol.* 18 287–295. 10.1177/039463200501800211 15888251

[B7] Alexandrakis M. G. Passam F. H. Pappa C. A. Sfiridaki K. Tsirakis G. Damilakis J. (2005). Relation between bone marrow angiogenesis and serum levels of angiogenin in patients with myelodysplastic syndromes. *Leuk. Res.* 29 41–46. 10.1016/j.leukres.2004.05.002 15541473

[B8] Ambrosi T. H. Scialdone A. Graja A. Gohlke S. Jank A.-M. Bocian C. (2017). Adipocyte accumulation in the bone marrow during obesity and aging impairs stem cell-based hematopoietic and bone regeneration. *Cell Stem Cell* 20 771–784.e6. 28330582 10.1016/j.stem.2017.02.009 PMC5459794

[B9] Ara T. Tokoyoda K. Sugiyama T. Egawa T. Kawabata K. Nagasawa T. (2003). Long-term hematopoietic stem cells require stromal cell-derived factor-1 for colonizing bone marrow during ontogeny. *Immunity* 19 257–267. 10.1016/s1074-7613(03)00201-2 12932359

[B10] Arai F. Hirao A. Ohmura M. Sato H. Matsuoka S. Takubo K. (2004). Tie2/angiopoietin-1 signaling regulates hematopoietic stem cell quiescence in the bone marrow niche. *Cell* 118 149–161. 10.1016/j.cell.2004.07.004 15260986

[B11] Arber D. A. Orazi A. Hasserjian R. Thiele J. Borowitz M. J. Beau M. M. L. (2016). The 2016 revision to the World Health Organization classification of myeloid neoplasms and acute leukemia. *Blood* 127 2391–2405. 10.1182/blood-2016-03-643544 27069254

[B12] Arranz L. Sánchez-Aguilera A. Martín-Pérez D. Isern J. Langa X. Tzankov A. (2014). Neuropathy of haematopoietic stem cell niche is essential for myeloproliferative neoplasms. *Nature* 512 78–81. 10.1038/nature13383 25043017

[B13] Asada N. Kunisaki Y. Pierce H. Wang Z. Fernandez N. F. Birbrair A. (2017). Differential cytokine contributions of perivascular haematopoietic stem cell niches. *Nat. Cell Biol.* 19 214–223. 10.1038/ncb3475 28218906 PMC5467892

[B14] Barbier V. Erbani J. Fiveash C. Davies J. M. Tay J. Tallack M. R. (2020). Endothelial E-selectin inhibition improves acute myeloid leukaemia therapy by disrupting vascular niche-mediated chemoresistance. *Nat. Commun.* 11:2042. 10.1038/s41467-020-15817-5 PMC7184728 32341362

[B15] Baryawno N. Przybylski D. Kowalczyk M. S. Kfoury Y. Severe N. Gustafsson K. (2019). A cellular taxonomy of the bone marrow stroma in homeostasis and leukemia. *Cell* 177 1915–1932.e16. 31130381 10.1016/j.cell.2019.04.040 PMC6570562

[B16] Beerman I. Rossi D. J. (2014). Epigenetic regulation of hematopoietic stem cell aging. *Exp. Cell Res.* 329 192–199. 10.1016/j.yexcr.2014.09.013 25261778 PMC4250347

[B17] Bellamy W. T. Richter L. Sirjani D. Roxas C. Glinsmann-Gibson B. Frutiger Y. (2001). Vascular endothelial cell growth factor is an autocrine promoter of abnormal localized immature myeloid precursors and leukemia progenitor formation in myelodysplastic syndromes. *Blood* 97 1427–1434. 10.1182/blood.v97.5.1427 11222390

[B18] Benito J. Ramirez M. S. Millward N. Z. Velez J. Harutyunyan K. G. Lu H. (2016). Hypoxia-activated prodrug TH-302 targets hypoxic bone marrow niches in preclinical leukemia models. *Clin. Cancer Res.* 22 1687–1698. 10.1158/1078-0432.ccr-14-3378 26603259 PMC4818660

[B19] Blau O. Baldus C. D. Hofmann W.-K. Thiel G. Nolte F. Burmeister T. (2011). Mesenchymal stromal cells of myelodysplastic syndrome and acute myeloid leukemia patients have distinct genetic abnormalities compared with leukemic blasts. *Blood* 118 5583–5592. 10.1182/blood-2011-03-343467 21948175 PMC3217359

[B20] Bloomgren G. Richman S. Hotermans C. Subramanyam M. Goelz S. Natarajan A. (2012). Risk of Natalizumab-associated progressive multifocal leukoencephalopathy. *N. Engl. J. Med.* 366 1870–1880. 22591293 10.1056/NEJMoa1107829

[B21] Bousse-Kerdilès M.-C. L. (2012). Primary myelofibrosis and the “bad seeds in bad soil” concept. *Fibrogenesis Tissue Repair* 5(Suppl. 1):S20. 10.1186/1755-1536-5-S1-S20 PMC3368798 23259918

[B22] Brunner B. Gunsilius E. Schumacher P. Zwierzina H. Gastl G. Stauder R. (2002). Blood levels of angiogenin and vascular endothelial growth factor are elevated in myelodysplastic syndromes and in acute myeloid leukemia. *J. Hematother. Stem Cell Res.* 11 119–125. 10.1089/152581602753448586 11847008

[B23] Bruns I. Lucas D. Pinho S. Ahmed J. Lambert M. P. Kunisaki Y. (2014). Megakaryocytes regulate hematopoietic stem cell quiescence through CXCL4 secretion. *Nat. Med.* 20 1315–1320. 10.1038/nm.3707 25326802 PMC4258871

[B24] Calvi L. M. Adams G. B. Weibrecht K. W. Weber J. M. Olson D. P. Knight M. C. (2003). Osteoblastic cells regulate the haematopoietic stem cell niche. *Nature* 425 841–846. 10.1038/nature02040 14574413

[B25] Cardier J. E. Barberá-Guillem E. (1997). Extramedullary hematopoiesis in the adult mouse liver is associated with specific hepatic sinusoidal endothelial cells. *Hepatology* 26 165–175. 10.1002/hep.510260122 9214466

[B26] Carrabba M. G. Casucci M. Hudecek M. Quintarelli C. Briones J. Hajek R. (2018). Phase I-IIa clinical trial to assess safety and efficacy of MLM-CAR44.1, a CD44v6 directed CAR-T in relapsed/refractory acute myeloid leukemia (AML) and multiple myeloma (MM) [Abstract]. *Blood* 132(Suppl. 1) 5790–5790. 10.1182/blood-2018-99-117974

[B27] Casanova-Acebes M. Pitaval C. Weiss L. A. Nombela-Arrieta C. Chèvre R. A-González N. (2013). Rhythmic modulation of the hematopoietic niche through neutrophil clearance. *Cell* 153 1025–1035. 10.1016/j.cell.2013.04.040 23706740 PMC4128329

[B28] Casella I. Feccia T. Chelucci C. Samoggia P. Castelli G. Guerriero R. (2003). Autocrine-paracrine VEGF loops potentiate the maturation of megakaryocytic precursors through Flt1 receptor. *Blood* 101 1316–1323. 10.1182/blood-2002-07-2184 12406876

[B29] Casucci M. Robilant B. N. D. Falcone L. Camisa B. Norelli M. Genovese P. (2013). CD44v6-targeted T cells mediate potent antitumor effects against acute myeloid leukemia and multiple myeloma. *Blood* 122 3461–3472. 10.1182/blood-2013-04-493361 24016461

[B30] Celso C. L. Fleming H. E. Wu J. W. Zhao C. X. Miake-Lye S. Fujisaki J. (2009). Live-animal tracking of individual haematopoietic stem/progenitor cells in their niche. *Nature* 457 92–96. 10.1038/nature07434 19052546 PMC2820276

[B31] Chaudhary A. K. Chaudhary S. Ghosh K. Shanmukaiah C. Nadkarni A. H. (2016). Secretion and expression of matrix metalloproteinase-2 and 9 from bone marrow mononuclear cells in myelodysplastic syndrome and acute myeloid leukemia. *Asian Pac. J. Cancer Prev.* 17 1519–1529. 10.7314/apjcp.2016.17.3.1519 27039800

[B32] Chen J. Y. Miyanishi M. Wang S. K. Yamazaki S. Sinha R. Kao K. S. (2016). Hoxb5 marks long-term haematopoietic stem cells and reveals a homogenous perivascular niche. *Nature* 530 223–227. 10.1038/nature16943 26863982 PMC4854608

[B33] Chen Q. Liu Y. Jeong H. W. Stehling M. Dinh V. V. Zhou B. (2019). Apelin(+) endothelial niche cells control hematopoiesis and mediate vascular regeneration after myeloablative injury. *Cell Stem Cell* 25 768–783 e6. 31761723 10.1016/j.stem.2019.10.006 PMC6900750

[B34] Cho B.-S. Kim H.-J. Konopleva M. (2017). Targeting the CXCL12/CXCR4 axis in acute myeloid leukemia: from bench to bedside. *Korean J. Intern. Med.* 32 248–257. 10.3904/kjim.2016.244 28219003 PMC5339474

[B35] Chow A. Lucas D. Hidalgo A. Méndez-Ferrer S. Hashimoto D. Scheiermann C. (2011). Bone marrow CD169^+^ macrophages promote the retention of hematopoietic stem and progenitor cells in the mesenchymal stem cell niche. *J. Exp. Med.* 208 261–271. 10.1084/jem.20101688 21282381 PMC3039855

[B36] Christodoulou C. Spencer J. A. Yeh S.-C. A. Turcotte R. Kokkaliaris K. D. Panero R. (2020). Live-animal imaging of native haematopoietic stem and progenitor cells. *Nature* 578 278–283. 10.1038/s41586-020-1971-z 32025033 PMC7021587

[B37] Cogle C. R. Bosse R. C. Brewer T. Migdady Y. Shirzad R. Kampen K. R. (2016). Acute myeloid leukemia in the vascular niche. *Cancer Lett.* 380 552–560. 10.1016/j.canlet.2015.05.007 25963886

[B38] Cogle C. R. Goldman D. C. Madlambayan G. J. Leon R. P. Masri A. A. Clark H. A. (2014). Functional integration of acute myeloid leukemia into the vascular niche. *Leukemia* 28 1978–1987. 10.1038/leu.2014.109 24637335 PMC4167983

[B39] Craver B. M. Alaoui K. E. Scherber R. M. Fleischman A. G. (2018). The critical role of inflammation in the pathogenesis and progression of myeloid malignancies. *Cancers* 10:104. 10.3390/cancers10040104 29614027 PMC5923359

[B40] DeAngelo D. J. Jonas B. A. Liesveld J. L. Bixby D. L. Advani A. S. Marlton P. (2018). Uproleselan (GMI-1271), an E-selectin antagonist, improves the efficacy and safety of chemotherapy in relapsed/refractory (R/R) and newly diagnosed older patients with acute myeloid leukemia: final, correlative, and subgroup analyses. *Blood* 132(Suppl. 1) 331–331. 10.1182/blood-2018-99-114286

[B41] Decker M. Leslie J. Liu Q. Ding L. (2018). Hepatic thrombopoietin is required for bone marrow hematopoietic stem cell maintenance. *Science* 360 106–110. 10.1126/science.aap8861 29622652 PMC5930357

[B42] Denkinger M. D. Leins H. Schirmbeck R. Florian M. C. Geiger H. (2015). HSC aging and senescent immune remodeling. *Trends Immunol.* 36 815–824. 10.1016/j.it.2015.10.008 26611154 PMC4710174

[B43] Dias S. Hattori K. Zhu Z. Heissig B. Choy M. Lane W. (2000). Autocrine stimulation of VEGFR-2 activates human leukemic cell growth and migration. *J. Clin. Invest.* 106 511–521. 10.1172/jci8978 10953026 PMC380247

[B44] DiNardo C. D. Stone R. M. Medeiros B. C. (2018). Novel therapeutics in acute myeloid leukemia. *Am. Soc. Clin. Oncol. Educ. Book* 37 495–503. 10.1200/EDBK_175401 28561688

[B45] Ding L. Morrison S. J. (2013). Haematopoietic stem cells and early lymphoid progenitors occupy distinct bone marrow niches. *Nature* 495 231–235. 10.1038/nature11885 23434755 PMC3600153

[B46] Ding L. Saunders T. L. Enikolopov G. Morrison S. J. (2012). Endothelial and perivascular cells maintain haematopoietic stem cells. *Nature* 481 457–462. 10.1038/nature10783 22281595 PMC3270376

[B47] Dominici M. Blanc K. L. Mueller I. Slaper-Cortenbach I. Marini F. C. Krause D. S. (2006). Minimal criteria for defining multipotent mesenchymal stromal cells. The international society for cellular therapy position statement. *Cytotherapy* 8 315–317. 10.1080/14653240600855905 16923606

[B48] Du Cheyne C. Tay H. De Spiegelaere W. (2020). The complex TIE between macrophages and angiogenesis. *Anat. Histol. Embryol.* 49 585–596. 31774212 10.1111/ahe.12518

[B49] Duarte D. Hawkins E. D. Akinduro O. Ang H. Filippo K. D. Kong I. Y. (2018a). Inhibition of endosteal vascular niche remodeling rescues hematopoietic stem cell loss in AML. *Cell Stem Cell* 22 64–77.e6. 29276143 10.1016/j.stem.2017.11.006 PMC5766835

[B50] Duarte D. Hawkins E. D. Celso C. L. (2018b). The interplay of leukemia cells and the bone marrow microenvironment. *Blood* 131 1507–1511. 10.1182/blood-2017-12-784132 29487069

[B51] Eliasson P. Rehn M. Hammar P. Larsson P. Sirenko O. Flippin L. A. (2010). Hypoxia mediates low cell-cycle activity and increases the proportion of long-term–reconstituting hematopoietic stem cells during in vitro culture. *Exp. Hematol.* 38 301.e–310.e. 301-310.e2, 20138114 10.1016/j.exphem.2010.01.005

[B52] Ellis S. L. Grassinger J. Jones A. Borg J. Camenisch T. Haylock D. (2011). The relationship between bone, hemopoietic stem cells, and vasculature. *Blood* 118 1516–1524. 10.1182/blood-2010-08-303800 21673348

[B53] Ergen A. V. Boles N. C. Goodell M. A. (2012). Rantes/Ccl5 influences hematopoietic stem cell subtypes and causes myeloid skewing. *Blood* 119 2500–2509. 10.1182/blood-2011-11-391730 22289892 PMC3311273

[B54] Fagiani E. Christofori G. (2013). Angiopoietins in angiogenesis. *Cancer Lett.* 328 18–26. 10.1016/j.canlet.2012.08.018 22922303

[B55] Fan L. Liu C. Hu A. Liang J. Li F. Xiong Y. (2020). Dual oligopeptides modification mediates arsenic trioxide containing nanoparticles to eliminate primitive chronic myeloid leukemia cells inside bone marrow niches. *Int. J. Pharmaceut.* 579 119179. 10.1016/j.ijpharm.2020.119179 32112927

[B56] Fang S. Chen S. Nurmi H. Leppänen V. M. Jeltsch M. Scadden D. (2020). VEGF-C protects the integrity of the bone marrow perivascular niche in mice. *Blood* 136 1871–1883. 10.1182/blood.2020005699 32842144 PMC7568034

[B57] Fantin A. Vieira J. M. Gestri G. Denti L. Schwarz Q. Prykhozhij S. (2010). Tissue macrophages act as cellular chaperones for vascular anastomosis downstream of VEGF-mediated endothelial tip cell induction. *Blood* 116 829–840. 10.1182/blood-2009-12-257832 20404134 PMC2938310

[B58] Feng X. Scheinberg P. Wu C. O. Samsel L. Nunez O. Prince C. (2011). Cytokine signature profiles in acquired aplastic anemia and myelodysplastic syndromes. *Haematologica* 96 602–606. 10.3324/haematol.2010.030536 21160069 PMC3069238

[B59] Fernandez L. Rodriguez S. Huang H. Chora A. Fernandes J. Mumaw C. (2008). Tumor necrosis factor-α and endothelial cells modulate Notch signaling in the bone marrow microenvironment during inflammation. *Exp. Hematol.* 36 545–558.e1. 18439488 10.1016/j.exphem.2007.12.012 PMC3437760

[B60] Fiedler W. Graeven U. Ergün S. Verago S. Kilic N. Stockschläder M. (1997). Vascular endothelial growth factor, a possible paracrine growth factor in human acute myeloid leukemia. *Blood* 89 1870–1875. 10.1182/blood.v89.6.1870 9058706

[B61] Fiedler W. Kayser S. Kebenko M. Janning M. Krauter J. Schittenhelm M. (2015). A phase I/II study of sunitinib and intensive chemotherapy in patients over 60 years of age with acute myeloid leukaemia and activating FLT3 mutations. *Br. J. Haematol.* 169 694–700. 10.1111/bjh.13353 25818407

[B62] Fiedler W. Mesters R. Tinnefeld H. Loges S. Staib P. Dührsen U. (2003). A phase 2 clinical study of SU5416 in patients with refractory acute myeloid leukemia. *Blood* 102 2763–2767. 12843001 10.1182/blood-2002-10-2998

[B63] Fiedler W. Serve H. Döhner H. Schwittay M. Ottmann O. G. O’Farrell A.-M. (2005). A phase 1 study of SU11248 in the treatment of patients with refractory or resistant acute myeloid leukemia (AML) or not amenable to conventional therapy for the disease. *Blood* 105 986–993. 10.1182/blood-2004-05-1846 15459012

[B64] Fiorentini A. Capelli D. Saraceni F. Menotti D. Poloni A. Olivieri A. (2020). The time has come for targeted therapies for AML: lights and shadows. *Oncol. Ther.* 8 13–32. 10.1007/s40487-019-00108-x 32700072 PMC7359996

[B65] Florian M. C. Dörr K. Niebel A. Daria D. Schrezenmeier H. Rojewski M. (2012). Cdc42 activity regulates hematopoietic stem cell aging and rejuvenation. *Cell Stem Cell* 10 520–530. 10.1016/j.stem.2012.04.007 22560076 PMC3348626

[B66] Folkes L. K. Christlieb M. Madej E. Stratford M. R. L. Wardman P. (2007). Oxidative metabolism of combretastatin A-1 produces quinone intermediates with the potential to bind to nucleophiles and to enhance oxidative stress via free radicals. *Chem. Res. Toxicol.* 20 1885–1894. 10.1021/tx7002195 17941699

[B67] Fong T. A. Shawver L. K. Sun L. Tang C. App H. Powell T. J. (1999). SU5416 is a potent and selective inhibitor of the vascular endothelial growth factor receptor (Flk-1/KDR) that inhibits tyrosine kinase catalysis, tumor vascularization, and growth of multiple tumor types. *Cancer Res.* 59 99–106. 9892193

[B68] Foss B. Ulvestad E. Bruserud Ø (2001). Platelet−derived growth factor (PDGF) in human acute myelogenous leukemia: PDGF receptor expression, endogenous PDGF release and responsiveness to exogenous PDGF isoforms by in vitro cultured acute myelogenous leukemia blasts. *Eur. J. Haematol.* 67 267–278. 10.1034/j.1600-0609.2001.0430a.x 11860452

[B69] Frisch B. J. Hoffman C. M. Latchney S. E. LaMere M. W. Myers J. Ashton J. (2019). Aged marrow macrophages expand platelet-biased hematopoietic stem cells via interleukin-1B. *JCI Insight* 4:e124213. 10.1172/jci.insight.124213 PMC6542605 30998506

[B70] Fujisaki J. Wu J. Carlson A. L. Silberstein L. Putheti P. Larocca R. (2011). In vivo imaging of Treg cells providing immune privilege to the haematopoietic stem-cell niche. *Nature* 474 216–219. 10.1038/nature10160 21654805 PMC3725645

[B71] Geiger H. Haan G. D. Florian M. C. (2013). The ageing haematopoietic stem cell compartment. *Nat. Rev. Immunol.* 13 376–389. 10.1038/nri3433 23584423

[B72] Geiger H. True J. M. Haan G. D. Zant G. V. (2001). Age- and stage-specific regulation patterns in the hematopoietic stem cell hierarchy. *Blood* 98 2966–2972. 10.1182/blood.v98.10.2966 11698278

[B73] Genovese G. Kähler A. K. Handsaker R. E. Lindberg J. Rose S. A. Bakhoum S. F. (2014). Clonal hematopoiesis and blood-cancer risk inferred from blood DNA sequence. *N. Engl. J. Med.* 371 2477–2487. 10.1056/nejmoa1409405 25426838 PMC4290021

[B74] Godavarthy P. S. Kumar R. Herkt S. C. Pereira R. S. Hayduk N. Weissenberger E. S. (2019). The vascular bone marrow niche influences outcome in chronic myeloid leukemia via the E-selectin - SCL/TAL1 - CD44 axis. *Haematologica* 105 136–147. 10.3324/haematol.2018.212365 31018977 PMC6939533

[B75] Gong J. K. (1978). Endosteal marrow: a rich source of hematopoietic stem cells. *Science* 199 1443–1445. 10.1126/science.75570 75570

[B76] Greenbaum A. Hsu Y.-M. S. Day R. B. Schuettpelz L. G. Christopher M. J. Borgerding J. N. (2013). CXCL12 in early mesenchymal progenitors is required for haematopoietic stem-cell maintenance. *Nature* 495 227–230. 10.1038/nature11926 23434756 PMC3600148

[B77] Guidi N. Sacma M. Ständker L. Soller K. Marka G. Eiwen K. (2017). Osteopontin attenuates aging−associated phenotypes of hematopoietic stem cells. *Embo J.* 36 840–853. 10.15252/embj.201694969 28254837 PMC5376966

[B78] Gupta P. Mulkey F. Hasserjian R. P. Sanford B. L. Vij R. Hurd D. D. (2013). A phase II study of the oral VEGF receptor tyrosine kinase inhibitor vatalanib (PTK787/ZK222584) in myelodysplastic syndrome: cancer and leukemia group B study 10105 (Alliance). *Invest. New Drug* 31 1311–1320. 10.1007/s10637-013-9978-z 23700288 PMC3773017

[B79] Hamberg P. Verweij J. Sleijfer S. (2010). (Pre−)Clinical pharmacology and Activity of Pazopanib, a Novel Multikinase Angiogenesis Inhibitor. *Oncologist* 15 539–547. 10.1634/theoncologist.2009-0274 20511320 PMC3227994

[B80] Hatfield K. Øyan A. M. Ersvaer E. Kalland K. H. Lassalle P. Gjertsen B. T. (2009). Primary human acute myeloid leukaemia cells increase the proliferation of microvascular endothelial cells through the release of soluble mediators. *Br. J. Haematol.* 144 53–68. 10.1111/j.1365-2141.2008.07411.x 19016730

[B81] Hazlehurst L. A. Dalton W. S. (2001). Mechanisms associated with cell adhesion mediated drug resistance (CAM-DR) in hematopoietic malignancies. *Cancer Metastasis. Rev.* 20 43–50. 11831646 10.1023/a:1013156407224

[B82] Hellström-Lindberg E. Tobiasson M. Greenberg P. (2020). Myelodysplastic syndromes: moving towards personalized management. *Haematologica* 105 1765–1779. 10.3324/haematol.2020.248955 32439724 PMC7327628

[B83] Hermitte F. Grange P.B.d.l Belloc F. Praloran V. Ivanovic Z. (2006). Very low O2 concentration (0.1%) favors G0 return of dividing CD34^+^ Cells. *Stem Cells* 24 65–73. 10.1634/stemcells.2004-0351 16123391

[B84] Hira V. V. V. Noorden C. J. F. V. Carraway H. E. Maciejewski J. P. Molenaar R. J. (2017). Novel therapeutic strategies to target leukemic cells that hijack compartmentalized continuous hematopoietic stem cell niches. *Biochim. Biophys. Acta* 1868 183–198. 10.1016/j.bbcan.2017.03.010 28363872

[B85] Ho Y.-H. Méndez-Ferrer S. (2019). Microenvironmental contributions to hematopoietic stem cell aging. *Haematologica* 105 38–46. 10.3324/haematol.2018.211334 31806690 PMC6939521

[B86] Ho Y.-H. Toro R. D. Rivera-Torres J. Rak J. Korn C. García-García A. (2019). Remodeling of bone marrow hematopoietic stem cell niches promotes myeloid cell expansion during premature or physiological aging. *Cell Stem Cell* 25 407–418.e6. 31303548 10.1016/j.stem.2019.06.007 PMC6739444

[B87] Holash J. Davis S. Papadopoulos N. Croll S. D. Ho L. Russell M. (2002). VEGF-Trap: a VEGF blocker with potent antitumor effects. *Proc. Natl. Acad Sci.* 99 11393–11398. 10.1073/pnas.172398299 12177445 PMC123267

[B88] Hooper A. T. Butler J. M. Nolan D. J. Kranz A. Iida K. Kobayashi M. (2009). Engraftment and reconstitution of hematopoiesis is dependent on VEGFR2-mediated regeneration of sinusoidal endothelial cells. *Cell Stem Cell* 4 263–274. 10.1016/j.stem.2009.01.006 19265665 PMC3228275

[B89] Hou H.-A. Chou W.-C. Lin L.-I. Tang J.-L. Tseng M.-H. Huang C.-F. (2008). Expression of angiopoietins and vascular endothelial growth factors and their clinical significance in acute myeloid leukemia. *Leuk. Res.* 32 904–912. 10.1016/j.leukres.2007.08.010 17904634

[B90] Hsieh Y.-T. Jiang E. Pham J. Kim H.-N. Abdel-Azim H. Khazal S. (2013). VLA4 blockade in acute myeloid leukemia [abstract]. *Blood* 122 3944–3944. 10.1182/blood.v122.21.3944.3944

[B91] Huizer K. Mustafa D. A. M. Spelt J. C. Kros J. M. Sacchetti A. (2017). Improving the characterization of endothelial progenitor cell subsets by an optimized FACS protocol. *PLoS One* 12:e0184895. 10.1371/journal.pone.0184895 28910385 PMC5599045

[B92] Hussong J. W. Rodgers G. M. Shami P. J. (2000). Evidence of increased angiogenesis in patients with acute myeloid leukemia. *Blood* 95 309–313. 10.1182/blood.v95.1.309.001k17_309_313 10607717

[B93] Ikeda H. Kanakura Y. Tamaki T. Kuriu A. Kitayama H. Ishikawa J. (1991). Expression and functional role of the proto-oncogene c-kit in acute myeloblastic leukemia cells. *Blood* 78 2962–2968. 10.1182/blood.v78.11.2962.bloodjournal78112962 1720040

[B94] Issa J.-P. J. (2013). The myelodysplastic syndrome as a prototypical epigenetic disease. *Blood* 121 3811–3817. 10.1182/blood-2013-02-451757 23660859 PMC3650703

[B95] Itkin T. Gur-Cohen S. Spencer J. A. Schajnovitz A. Ramasamy S. K. Kusumbe A. P. (2016). Distinct bone marrow blood vessels differentially regulate haematopoiesis. *Nature* 532 323–328. 10.1038/nature17624 27074509 PMC6450701

[B96] Iwata M. Pillai M. Ramakrishnan A. Hackman R. C. Deeg H. J. Opdenakker G. (2007). Reduced expression of inducible gelatinase B/matrix metalloproteinase-9 in monocytes from patients with myelodysplastic syndrome: correlation of inducible levels with the percentage of cytogenetically marked cells and with marrow cellularity. *Blood* 109 85–92. 10.1182/blood-2006-05-020289 16954500 PMC1785081

[B97] Jacamo R. Chen Y. Wang Z. Ma W. Zhang M. Spaeth E. L. (2014). Reciprocal leukemia-stroma VCAM-1/VLA-4-dependent activation of NF-κB mediates chemoresistance. *Blood* 123 2691–2702. 10.1182/blood-2013-06-511527 24599548 PMC3999754

[B98] Jagannathan-Bogdan M. Zon L. I. (2013). Hematopoiesis. *Development* 140 2463–2467. 23715539 10.1242/dev.083147 PMC3666375

[B99] Jaiswal S. Fontanillas P. Flannick J. Manning A. Grauman P. V. Mar B. G. (2014). Age-related clonal hematopoiesis associated with adverse outcomes. *N. Engl. J. Med.* 371 2488–2498. 25426837 10.1056/NEJMoa1408617 PMC4306669

[B100] Jaiswal S. Natarajan P. Silver A. J. Gibson C. J. Bick A. G. Shvartz E. (2017). Clonal hematopoiesis and risk of atherosclerotic cardiovascular disease. *N. Engl. J. Med.* 377 111–121. 28636844 10.1056/NEJMoa1701719 PMC6717509

[B101] Ji Y.-T. Liu Y.-N. Liu Z.-P. (2015). Tubulin colchicine binding site inhibitors as vascular disrupting agents in clinical developments. *Curr. Med. Chem.* 22 1348–1360. 10.2174/0929867322666150114163732 25620094

[B102] Jin L. Hope K. J. Zhai Q. Smadja-Joffe F. Dick J. E. (2006). Targeting of CD44 eradicates human acute myeloid leukemic stem cells. *Nat. Med.* 12 1167–1174. 10.1038/nm1483 16998484

[B103] Kampen K. R. Elst A. T. de Bont E. S. (2013). Vascular endothelial growth factor signaling in acute myeloid leukemia. *Cell. Mol Life Sci* 70 1307–1317. 10.1007/s00018-012-1085-3 22833169 PMC11113417

[B104] Karp J. E. Gojo I. Pili R. Gocke C. D. Greer J. Guo C. (2004). Targeting vascular endothelial growth factor for relapsed and refractory adult acute myelogenous leukemias therapy with sequential 1-β-d-arabinofuranosylcytosine, mitoxantrone, and bevacizumab. *Clin. Cancer Res.* 10 3577–3585. 10.1158/1078-0432.ccr-03-0627 15173063

[B105] Katayama Y. Battista M. Kao W.-M. Hidalgo A. Peired A. J. Thomas S. A. (2006). Signals from the sympathetic nervous system regulate hematopoietic stem cell egress from bone marrow. *Cell* 124 407–421. 10.1016/j.cell.2005.10.041 16439213

[B106] Kessler T. Koschmieder S. Schliemann C. Crysandt M. Mikesch J.-H. von Stillfried S. (2019). Phase II clinical trial of pazopanib in patients with acute myeloid leukemia (AML), relapsed or refractory or at initial diagnosis without an intensive treatment option (PazoAML). *Ann. Hematol.* 98 1393–1401. 10.1007/s00277-019-03651-9 30903275

[B107] Kiel M. J. Yilmaz ÖH. Iwashita T. Yilmaz O. H. Terhorst C. Morrison S. J. (2005). SLAM family receptors distinguish hematopoietic stem and progenitor cells and reveal endothelial niches for stem cells. *Cell* 121 1109–1121. 10.1016/j.cell.2005.05.026 15989959

[B108] Kim C. K. Han D. H. Ji Y. S. Lee M. S. Min C. W. Park S. K. (2016). Biomarkers of angiogenesis as prognostic factors in myelodysplastic syndrome patients treated with hypomethylating agents. *Leuk. Res.* 50 21–28. 10.1016/j.leukres.2016.08.012 27639703

[B109] Kim Y.-W. Koo B.-K. Jeong H.-W. Yoon M.-J. Song R. Shin J. (2008). Defective notch activation in microenvironment leads to myeloproliferative disease. *Blood* 112 4628–4638. 10.1182/blood-2008-03-148999 18818392

[B110] Kirschbaum M. H. Frankel P. Synold T. W. Zain J. Claxton D. Tuscano J. (2018). A phase II study of vascular endothelial growth factor trap (Aflibercept, NSC 724770) in patients with myelodysplastic syndrome: a California cancer consortium study. *Br. J. Haematol.* 180 445–448. 10.1111/bjh.14333 27650362 PMC5360527

[B111] Kode A. Manavalan J. S. Mosialou I. Bhagat G. Rathinam C. V. Luo N. (2014). Leukaemogenesis induced by an activating β-catenin mutation in osteoblasts. *Nature* 506 240–244. 10.1038/nature12883 24429522 PMC4116754

[B112] Köhler A. Schmithorst V. Filippi M.-D. Ryan M. A. Daria D. Gunzer M. (2009). Altered cellular dynamics and endosteal location of aged early hematopoietic progenitor cells revealed by time-lapse intravital imaging in long bones. *Blood* 114 290–298. 10.1182/blood-2008-12-195644 19357397 PMC2714205

[B113] Kokkaliaris K. Kunz L. Cabezas-Wallscheid N. Christodoulou C. Renders S. Camargo F. (2020). Adult blood stem cell localization reflects the abundance of reported bone marrow niche cell types and their combinations. *Blood* 136 2296–2307. 10.1182/blood.2020006574 32766876 PMC8209553

[B114] Korkolopoulou P. Apostolidou E. Pavlopoulos P. M. Kavantzas N. Vyniou N. Thymara I. (2001). Prognostic evaluation of the microvascular network in myelodysplastic syndromes. *Leukemia* 15 1369–1376. 10.1038/sj.leu.2402220 11516097

[B115] Korolnek T. Hamza I. (2015). Macrophages and iron trafficking at the birth and death of red cells. *Blood* 125 2893–2897. 10.1182/blood-2014-12-567776 25778532 PMC4424413

[B116] Kovtonyuk L. V. Fritsch K. Feng X. Manz M. G. Takizawa H. (2016). Inflamm-aging of hematopoiesis, hematopoietic stem cells, and the bone marrow microenvironment. *Front. Immunol.* 7:502. 10.3389/fimmu.2016.00502 27895645 PMC5107568

[B117] Kricun M. E. (1985). Red-yellow marrow conversion: its effect on the location of some solitary bone lesions. *Skeletal Radiol.* 14 10–19. 10.1007/bf00361188 3895447

[B118] Kumar B. Chen C. C. (2018). Acute myeloid leukemia remodels endosteal vascular niche into a leukemic niche. *Stem Cell Investig.* 5 34–34. 10.21037/sci.2018.09.05 30498745 PMC6232068

[B119] Kunisaki Y. Bruns I. Scheiermann C. Ahmed J. Pinho S. Zhang D. (2013). Arteriolar niches maintain haematopoietic stem cell quiescence. *Nature* 502 637–643. 10.1038/nature12612 24107994 PMC3821873

[B120] Kuribayashi W. Iwama A. Oshima M. (2019). Incomplete rejuvenation of aged HSCs in young bone marrow niche [abstract]. *Exp. Hematol.* 76:S72.

[B121] Kusumbe A. P. Ramasamy S. K. Adams R. H. (2014). Coupling of angiogenesis and osteogenesis by a specific vessel subtype in bone. *Nature* 507 323–328. 10.1038/nature13145 24646994 PMC4943525

[B122] Kusumbe A. P. Ramasamy S. K. Itkin T. Mäe M. A. Langen U. H. Betsholtz C. (2016). Age-dependent modulation of vascular niches for haematopoietic stem cells. *Nature* 532 380–384. 10.1038/nature17638 27074508 PMC5035541

[B123] Kuzu I. Beksac M. Arat M. Celebi H. Elhan A. H. Erekul S. (2009). Bone marrow microvessel density (MVD) in adult acute myeloid leukemia (AML): therapy induced changes and effects on survival. *Leuk. Lymphoma* 45 1185–1190. 10.1080/1042819032000159915 15359999

[B124] Lal D. Park J. A. Demock K. Marinaro J. Perez A. M. Lin M.-H. (2010). Aflibercept exerts antivascular effects and enhances levels of anthracycline chemotherapy in vivo in human acute myeloid leukemia models. *Mol. Cancer Ther.* 9 2737–2751. 10.1158/1535-7163.mct-10-0334 20924124

[B125] Lataillade J.-J. Pierre-Louis O. Hasselbalch H. C. Uzan G. Jasmin C. Martyré M.-C. (2008). Does primary myelofibrosis involve a defective stem cell niche? From concept to evidence. *Blood* 112 3026–3035. 10.1182/blood-2008-06-158386 18669872

[B126] Layani-Bazar A. Skornick I. Berrebi A. Pauker M. H. Noy E. Silberman A. (2014). Redox modulation of adjacent thiols in VLA-4 by AS101 converts myeloid leukemia cells from a drug-resistant to drug-sensitive state. *Cancer Res.* 74 3092–3103. 10.1158/0008-5472.can-13-2159 24699624

[B127] Li W. Johnson S. A. Shelley W. C. Yoder M. C. (2004). Hematopoietic stem cell repopulating ability can be maintained in vitro by some primary endothelial cells. *Exp. Hematol.* 32 1226–1237. 10.1016/j.exphem.2004.09.001 15588947

[B128] Li X. Zeng X. Xu Y. Wang B. Zhao Y. Lai X. (2020). Mechanisms and rejuvenation strategies for aged hematopoietic stem cells. *J. Hematol. Oncol.* 13:31. 10.1186/s13045-020-00864-8 PMC7137344 32252797

[B129] Libby P. Sidlow R. Lin A. E. Gupta D. Jones L. W. Moslehi J. (2019). Clonal hematopoiesis crossroads of aging, cardiovascular disease, and cancer: JACC review topic of the week. *J. Am. Coll. Cardiol.* 74 567–577. 31345432 10.1016/j.jacc.2019.06.007 PMC6681657

[B130] List A. F. Glinsmann-Gibson B. Stadheim C. Meuillet E. J. Bellamy W. Powis G. (2004). Vascular endothelial growth factor receptor-1 and receptor-2 initiate a phosphatidylinositide 3-kinase–dependent clonogenic response in acute myeloid leukemia cells. *Exp. Hematol.* 32 526–535. 10.1016/j.exphem.2004.03.005 15183893

[B131] Loges S. Heil G. Bruweleit M. Schoder V. Butzal M. Fischer U. (2005). Analysis of concerted expression of angiogenic growth factors in acute myeloid leukemia: expression of angiopoietin-2 represents an independent prognostic factor for overall survival. *J. Clin. Oncol.* 23 1109–1117. 10.1200/jco.2005.05.058 15718307

[B132] Lord B. I. Testa N. G. Hendry J. H. (1975). The relative spatial distributions of CFUs and CFUc in the normal mouse femur. *Blood* 46 65–72. 10.1182/blood.v46.1.65.65 1131427

[B133] Lucas D. Scheiermann C. Chow A. Kunisaki Y. Bruns I. Barrick C. (2013). Chemotherapy-induced bone marrow nerve injury impairs hematopoietic regeneration. *Nat. Med.* 19 695–703. 10.1038/nm.3155 23644514 PMC3964478

[B134] Madlambayan G. J. Meacham A. M. Hosaka K. Mir S. Jorgensen M. Scott E. W. (2010). Leukemia regression by vascular disruption and antiangiogenic therapy. *Blood* 116 1539–1547. 10.1182/blood-2009-06-230474 20472832 PMC2938842

[B135] Maganti H. Visram A. Shorr R. Fulcher J. Sabloff M. Allan D. S. (2020). Plerixafor in combination with chemotherapy and/or hematopoietic cell transplantation to treat acute leukemia: a systematic review and metanalysis of preclinical and clinical studies. *Leuk. Res.* 97:106442. 10.1016/j.leukres.2020.106442 32877869

[B136] Mangi M. H. Salisbury J. R. Mufti G. J. (1991). Abnormal localization of immature precursors (ALIP) in the bone marrow of myelodysplastic syndromes: current state of knowledge and future directions. *Leuk. Res.* 15 627–639. 10.1016/0145-2126(91)90032-o 1861544

[B137] Maryanovich M. Zahalka A. H. Pierce H. Pinho S. Nakahara F. Asada N. (2018). Adrenergic nerve degeneration in bone marrow drives aging of the hematopoietic stem cell niche. *Nat. Med.* 24 782–791. 10.1038/s41591-018-0030-x 29736022 PMC6095812

[B138] McKerrell T. Park N. Moreno T. Grove C. S. Ponstingl H. Stephens J. (2015). Leukemia-associated somatic mutations drive distinct patterns of age-related clonal hemopoiesis. *Cell Rep.* 10 1239–1245. 10.1016/j.celrep.2015.02.005 25732814 PMC4542313

[B139] Medyouf H. Mossner M. Jann J.-C. Nolte F. Raffel S. Herrmann C. (2014). Myelodysplastic cells in patients reprogram mesenchymal stromal cells to establish a transplantable stem cell niche disease unit. *Cell Stem Cell* 14 824–837. 10.1016/j.stem.2014.02.014 24704494

[B140] Méndez-Ferrer S. Lucas D. Battista M. Frenette P. S. (2008). Haematopoietic stem cell release is regulated by circadian oscillations. *Nature* 452 442–447. 10.1038/nature06685 18256599

[B141] Méndez-Ferrer S. Michurina T. V. Ferraro F. Mazloom A. R. MacArthur B. D. Lira S. A. (2010). Mesenchymal and haematopoietic stem cells form a unique bone marrow niche. *Nature* 466 829–834. 10.1038/nature09262 20703299 PMC3146551

[B142] Mizoguchi T. Pinho S. Ahmed J. Kunisaki Y. Hanoun M. Mendelson A. (2014). Osterix marks distinct waves of primitive and definitive stromal progenitors during bone marrow development. *Dev. Cell* 29 340–349. 10.1016/j.devcel.2014.03.013 24823377 PMC4051418

[B143] Morrison S. J. Wandycz A. M. Akashi K. Globerson A. Weissman I. L. (1996). The aging of hematopoietic stem cells. *Nat. Med.* 2 1011–1016. 8782459 10.1038/nm0996-1011

[B144] Moukalled N. M. Rassi F. A. E. Temraz S. N. Taher A. T. (2018). Iron overload in patients with myelodysplastic syndromes: an updated overview. *Cancer* 124 3979–3989. 10.1002/cncr.31550 29905937

[B145] Müller A. Lange K. Gaiser T. Hofmann M. Bartels H. Feller A. C. (2002). Expression of angiopoietin-1 and its receptor TEK in hematopoietic cells from patients with myeloid leukemia. *Leuk. Res.* 26 163–168. 10.1016/s0145-2126(01)00110-2 11755466

[B146] Nakamura-Ishizu A. Takubo K. Fujioka M. Suda T. (2014). Megakaryocytes are essential for HSC quiescence through the production of thrombopoietin. *Biochem. Biophs. Res. Commun.* 454 353–357. 10.1016/j.bbrc.2014.10.095 25451253

[B147] Naveiras O. Nardi V. Wenzel P. L. Hauschka P. V. Fahey F. Daley G. Q. (2009). Bone-marrow adipocytes as negative regulators of the haematopoietic microenvironment. *Nature* 460 259–263. 10.1038/nature08099 19516257 PMC2831539

[B148] Nilsson S. K. Johnston H. M. Whitty G. A. Williams B. Webb R. J. Denhardt D. T. (2005). Osteopontin, a key component of the hematopoietic stem cell niche and regulator of primitive hematopoietic progenitor cells. *Blood* 106 1232–1239. 10.1182/blood-2004-11-4422 15845900

[B149] Niyongere S. Lucas N. Zhou J.-M. Sansil S. Pomicter A. D. Balasis M. E. (2018). Heterogeneous expression of cytokines accounts for clinical diversity and refines prognostication in CMML. *Leukemia* 33 205–216. 10.1038/s41375-018-0203-0 30026572 PMC7787307

[B150] Nombela-Arrieta C. Pivarnik G. Winkel B. Canty K. J. Harley B. Mahoney J. E. (2013). Quantitative imaging of haematopoietic stem and progenitor cell localization and hypoxic status in the bone marrow microenvironment. *Nat. Cell Biol.* 15 533–543. 10.1038/ncb2730 23624405 PMC4156024

[B151] Nybakken G. Gratzinger D. (2016). Myelodysplastic syndrome macrophages have aberrant iron storage and heme oxygenase-1 expression. *Leuk. Lymphoma* 57 1893–1902. 26758041 10.3109/10428194.2015.1121259

[B152] O’Farrell A.-M. Abrams T. J. Yuen H. A. Ngai T. J. Louie S. G. Yee K. W. H. (2003). SU11248 is a novel FLT3 tyrosine kinase inhibitor with potent activity in vitro and in vivo. *Blood* 101 3597–3605. 10.1182/blood-2002-07-2307 12531805

[B153] Ogawa M. Matsuzaki Y. Nishikawa S. Hayashi S. Kunisada T. Sudo T. (1991). Expression and function of c-kit in hemopoietic progenitor cells. *J. Exp. Med.* 174 63–71. 10.1084/jem.174.1.63 1711568 PMC2118893

[B154] Ohneda O. Fennie C. Zheng Z. Donahue C. La H. Villacorta R. (1998). Hematopoietic stem cell maintenance and differentiation are supported by embryonic aorta-gonad-mesonephros region–derived endothelium. *Blood* 92 908–919. 10.1182/blood.v92.3.908.415k13_908_919 9680359

[B155] Omatsu Y. Sugiyama T. Kohara H. Kondoh G. Fujii N. Kohno K. (2010). The essential functions of adipo-osteogenic progenitors as the hematopoietic stem and progenitor cell niche. *Immunity* 33 387–399. 10.1016/j.immuni.2010.08.017 20850355

[B156] Ono N. Ono W. Mizoguchi T. Nagasawa T. Frenette P. S. Kronenberg H. M. (2014). Vasculature-associated cells expressing nestin in developing bones encompass early cells in the osteoblast and endothelial lineage. *Dev. Cell* 29 330–339. 10.1016/j.devcel.2014.03.014 24823376 PMC4083679

[B157] Ossenkoppele G. J. Stussi G. Maertens J. van Montfort K. Biemond B. J. Breems D. (2012). Addition of bevacizumab to chemotherapy in acute myeloid leukemia at older age: a randomized phase 2 trial of the dutch-belgian cooperative trial group for hemato-oncology (HOVON) and the swiss group for clinical cancer research (SAKK). *Blood* 120 4706–4711. 10.1182/blood-2012-04-420596 23047822

[B158] Ottersbach K. (2019). Endothelial-to-haematopoietic transition: an update on the process of making blood. *Biochem. Soc. Trans.* 47:BST20180320. 10.1042/BST20180320 PMC6490701 30902922

[B159] Padró T. Bieker R. Ruiz S. Steins M. Retzlaff S. Bürger H. (2002). Overexpression of vascular endothelial growth factor (VEGF) and its cellular receptor KDR (VEGFR-2) in the bone marrow of patients with acute myeloid leukemia. *Leukemia* 16 1302–1310. 10.1038/sj.leu.2402534 12094254

[B160] Padró T. Ruiz S. Bieker R. Bürger H. Steins M. Kienast J. (2000). Increased angiogenesis in the bone marrow of patients with acute myeloid leukemia. *Blood* 95 2637–2644. 10753845

[B161] Pardanani A. Finke C. Lasho T. L. Al-Kali A. Begna K. H. Hanson C. A. (2012). IPSS-independent prognostic value of plasma CXCL10, IL-7 and IL-6 levels in myelodysplastic syndromes. *Leukemia* 26 693–699. 10.1038/leu.2011.251 21912394 PMC3364441

[B162] Park M. H. Jin H. K. Min W. K. Lee W. W. Lee J. E. Akiyama H. (2015). Neuropeptide Y regulates the hematopoietic stem cell microenvironment and prevents nerve injury in the bone marrow. *Embo J.* 34 1648–1660. 10.15252/embj.201490174 25916827 PMC4475399

[B163] Parmar K. Mauch P. Vergilio J.-A. Sackstein R. Down J. D. (2007). Distribution of hematopoietic stem cells in the bone marrow according to regional hypoxia. *Proc. Natl. Acad Sci.* 104 5431–5436. 10.1073/pnas.0701152104 17374716 PMC1838452

[B164] Passaro D. Tullio A. D. Abarrategi A. Rouault-Pierre K. Foster K. Ariza-McNaughton L. (2017). Increased vascular permeability in the bone marrow microenvironment contributes to disease progression and drug response in acute myeloid leukemia. *Cancer Cell* 32 324–341.e6. 28870739 10.1016/j.ccell.2017.08.001 PMC5598545

[B165] Petit I. Karajannis M. A. Vincent L. Young L. Butler J. Hooper A. T. (2008). The microtubule-targeting agent CA4P regresses leukemic xenografts by disrupting interaction with vascular cells and mitochondrial-dependent cell death. *Blood* 111 1951–1961. 10.1182/blood-2007-05-089219 18024794 PMC2234044

[B166] Petit I. Szyper-Kravitz M. Nagler A. Lahav M. Peled A. Habler L. (2002). G-CSF induces stem cell mobilization by decreasing bone marrow SDF-1 and up-regulating CXCR4. *Nat. Immunol.* 3 687–694. 10.1038/ni813 12068293

[B167] Phinney D. G. Giuseppe M. D. Njah J. Sala E. Shiva S. Croix C. M. S. (2015). Mesenchymal stem cells use extracellular vesicles to outsource mitophagy and shuttle microRNAs. *Nat. Commun.* 6:8472. 10.1038/ncomms9472 PMC4598952 26442449

[B168] Pietras E. M. (2017). Inflammation: a key regulator of hematopoietic stem cell fate in health and disease. *Blood* 130 1693–1698. 10.1182/blood-2017-06-780882 28874349 PMC5639485

[B169] Pinho S. Frenette P. S. (2019). Haematopoietic stem cell activity and interactions with the niche. *Nat. Rev. Mol. Cell Biol.* 20 303–320. 10.1038/s41580-019-0103-9 30745579 PMC6483843

[B170] Pinho S. Lacombe J. Hanoun M. Mizoguchi T. Bruns I. Kunisaki Y. (2013). PDGFRα and CD51 mark human Nestin+ sphere-forming mesenchymal stem cells capable of hematopoietic progenitor cell expansionNestin+ MSCs in the human bone marrow. *J. Exp. Med.* 210 1351–1367. 10.1084/jem.20122252 23776077 PMC3698522

[B171] Polverini P. J. Cotran P. S. Gimbrone M. A. Unanue E. R. (1977). Activated macrophages induce vascular proliferation. *Nature* 269 804–806. 10.1038/269804a0 927505

[B172] Porta M. G. D. Malcovati L. Rigolin G. M. Rosti V. Bonetti E. Travaglino E. (2008). Immunophenotypic, cytogenetic and functional characterization of circulating endothelial cells in myelodysplastic syndromes. *Leukemia* 22 530–537. 10.1038/sj.leu.2405069 18094717

[B173] Poulos M. G. Guo P. Kofler N. M. Pinho S. Gutkin M. C. Tikhonova A. (2013). Endothelial jagged-1 Is necessary for homeostatic and regenerative hematopoiesis. *Cell Rep.* 4 1022–1034. 10.1016/j.celrep.2013.07.048 24012753 PMC3805263

[B174] Poulos M. G. Ramalingam P. Gutkin M. C. Kleppe M. Ginsberg M. Crowley M. J. P. (2016). Endothelial-specific inhibition of NF-κB enhances functional haematopoiesis. *Nat. Commun.* 7:13829. 10.1038/ncomms13829 PMC5187502 28000664

[B175] Poulos M. G. Ramalingam P. Gutkin M. C. Llanos P. Gilleran K. Rabbany S. Y. (2017). Endothelial transplantation rejuvenates aged hematopoietic stem cell function. *J. Clin. Invest.* 127 4163–4178. 10.1172/jci93940 29035282 PMC5663355

[B176] Poussin C. Gallitz I. Schlage W. K. Steffen Y. Stolle K. Lebrun S. (2014). Mechanism of an indirect effect of aqueous cigarette smoke extract on the adhesion of monocytic cells to endothelial cells in an in vitro assay revealed by transcriptomics analysis. *Toxicol. In Vitro* 28 896–908. 10.1016/j.tiv.2014.03.005 24747719

[B177] Pruneri G. Bertolini F. Soligo D. Carboni N. Cortelezzi A. Ferrucci P. F. (1999). Angiogenesis in myelodysplastic syndromes. *Br. J. Cancer* 81 1398–1401. 10604739 10.1038/sj.bjc.6693515 PMC2362976

[B178] Qian H. Buza-Vidas N. Hyland C. D. Jensen C. T. Antonchuk J. Månsson R. (2007). Critical role of thrombopoietin in maintaining adult quiescent hematopoietic stem cells. *Cell Stem Cell* 1 671–684. 10.1016/j.stem.2007.10.008 18371408

[B179] Raaijmakers M. H. G. P. Mukherjee S. Guo S. Zhang S. Kobayashi T. Schoonmaker J. A. (2010). Bone progenitor dysfunction induces myelodysplasia and secondary leukaemia. *Nature* 464 852–857. 10.1038/nature08851 20305640 PMC3422863

[B180] Ramalingam P. Poulos M. G. Butler J. M. (2017). Regulation of the hematopoietic stem cell lifecycle by the endothelial niche. *Curr. Opin. Hematol.* 24 289–299. 10.1097/moh.0000000000000350 28594660 PMC5554937

[B181] Ramalingam P. Poulos M. G. Gutkin M. C. Katsnelson L. Freire A. G. Lazzari E. (2020). Endothelial mTOR maintains hematopoiesis during aging. *J. Exp. Med.* 217:e20191212. 10.1084/jem.20191212 PMC7971143 32289154

[B182] Ramasamy S. K. Kusumbe A. P. Schiller M. Zeuschner D. Bixel M. G. Milia C. (2016). Blood flow controls bone vascular function and osteogenesis. *Nat. Commun.* 7:13601. 10.1038/ncomms13601 PMC5150650 27922003

[B183] Ramasamy S. K. Kusumbe A. P. Wang L. Adams R. H. (2014). Endothelial Notch activity promotes angiogenesis and osteogenesis in bone. *Nature* 507 376–380. 10.1038/nature13146 24647000 PMC4943529

[B184] Reikvam H. Hatfield K. J. Lassalle P. Kittang A. O. Ersvær E. Bruserud Ø (2010). Targeting the angiopoietin (Ang)/Tie-2 pathway in the crosstalk between acute myeloid leukaemia and endothelial cells: studies of Tie-2 blocking antibodies, exogenous Ang-2 and inhibition of constitutive agonistic Ang-1 release. *Exp. Opin. Inv. Drug* 19 169–183. 10.1517/13543780903485659 20050812

[B185] Richardson D. S. Lichtman J. W. (2015). Clarifying tissue clearing. *Cell* 162 246–257. 10.1016/j.cell.2015.06.067 26186186 PMC4537058

[B186] Roboz G. J. Giles F. J. List A. F. Cortes J. E. Carlin R. Kowalski M. (2006). Phase 1 study of PTK787/ZK 222584, a small molecule tyrosine kinase receptor inhibitor, for the treatment of acute myeloid leukemia and myelodysplastic syndrome. *Leukemia* 20 952–957. 10.1038/sj.leu.2404213 16617323

[B187] Roskoski R. (2007). Sunitinib: a VEGF and PDGF receptor protein kinase and angiogenesis inhibitor. *Biochem. Biophys. Res. Commun.* 356 323–328. 10.1016/j.bbrc.2007.02.156 17367763

[B188] Rossi D. J. Bryder D. Zahn J. M. Ahlenius H. Sonu R. Wagers A. J. (2005). Cell intrinsic alterations underlie hematopoietic stem cell aging. *Proc. Natl. Acad. Sci. U.S.A.* 102 9194–9199. 10.1073/pnas.0503280102 15967997 PMC1153718

[B189] Rozhok A. I. Salstrom J. L. DeGregori J. (2014). Stochastic modeling indicates that aging and somatic evolution in the hematopoietic system are driven by non-cell-autonomous processes. *Aging* 6 1033–1048. 10.18632/aging.100707 25564763 PMC4298364

[B190] Ruffini P. A. (2019). The CXCL8-CXCR1/2 axis as a therapeutic target in breast cancer stem-like cells. *Front. Oncol.* 9:40. 10.3389/fonc.2019.00040 30788286 PMC6373429

[B191] Savic A. Cemerikic-Martinovic V. Dovat S. Rajic N. Urosevic I. Sekulic B. (2012). Angiogenesis and survival in patients with myelodysplastic syndrome. *Pathol. Oncol. Res.* 18 681–690. 10.1007/s12253-012-9495-y 22270865

[B192] Schliemann C. Bieker R. Padro T. Kessler T. Hintelmann H. Buchner T. (2006). Expression of angiopoietins and their receptor Tie2 in the bone marrow of patients with acute myeloid leukemia. *Haematologica* 91 1203–1211. 16956819

[B193] Schliemann C. Bieker R. Thoennissen N. Gerss J. Liersch R. Kessler T. (2007). Circulating angiopoietin-2 is a strong prognostic factor in acute myeloid leukemia. *Leukemia* 21 1901–1906. 10.1038/sj.leu.2404820 17597808

[B194] Severe N. Karabacak N. M. Gustafsson K. Baryawno N. Courties G. Kfoury Y. (2019). Stress-induced changes in bone marrow stromal cell populations revealed through single-cell protein expression mapping. *Cell Stem Cell* 25 570–583.e7. 31279774 10.1016/j.stem.2019.06.003 PMC6778015

[B195] Seyfried A. N. Maloney J. M. MacNamara K. C. (2020). Macrophages orchestrate hematopoietic programs and regulate HSC function during inflammatory stress. *Front. Immunol.* 11:1499. 10.3389/fimmu.2020.01499 32849512 PMC7396643

[B196] Shi X. Zheng Y. Xu L. Cao C. Dong B. Chen X. (2019). The inflammatory cytokine profile of myelodysplastic syndromes: a meta-analysis. *Medicine (Baltimore)* 98:e15844. 10.1097/md.0000000000015844 31145332 PMC6708708

[B197] Silberstein L. Goncalves K. A. Kharchenko P. V. Turcotte R. Kfoury Y. Mercier F. (2016). Proximity-based differential single-cell analysis of the niche to identify stem/progenitor cell regulators. *Cell Stem Cell* 19 530–543. 10.1016/j.stem.2016.07.004 27524439 PMC5402355

[B198] Skinner A. M. Grompe M. Kurre P. (2012). Intra-hematopoietic cell fusion as a source of somatic variation in the hematopoietic system. *J. Cell Sci.* 125 2837–2843. 10.1242/jcs.100123 22393240 PMC3434805

[B199] Smolich B. D. Yuen H. A. West K. A. Giles F. J. Albitar M. Cherrington J. M. (2001). The antiangiogenic protein kinase inhibitors SU5416 and SU6668 inhibit the SCF receptor (c-kit) in a human myeloid leukemia cell line and in acute myeloid leukemia blasts. *Blood* 97 1413–1421. 10.1182/blood.v97.5.1413 11222388

[B200] Spencer J. A. Ferraro F. Roussakis E. Klein A. Wu J. Runnels J. M. (2014). Direct measurement of local oxygen concentration in the bone marrow of live animals. *Nature* 508 269–273. 10.1038/nature13034 24590072 PMC3984353

[B201] Steensma D. P. Bejar R. Jaiswal S. Lindsley R. C. Sekeres M. A. Hasserjian R. P. (2015). Clonal hematopoiesis of indeterminate potential and its distinction from myelodysplastic syndromes. *Blood* 126 9–16. 10.1182/blood-2015-03-631747 25931582 PMC4624443

[B202] Stier S. Ko Y. Forkert R. Lutz C. Neuhaus T. Grünewald E. (2005). Osteopontin is a hematopoietic stem cell niche component that negatively regulates stem cell pool size. *J. Exp. Med.* 201 1781–1791. 10.1084/jem.20041992 15928197 PMC2213260

[B203] Stucki A. Rivier A.-S. Gikic M. Monai N. Schapira M. Spertini O. (2001). Endothelial cell activation by myeloblasts: molecular mechanisms of leukostasis and leukemic cell dissemination. *Blood* 97 2121–2129. 10.1182/blood.v97.7.2121 11264180

[B204] Sugiyama T. Kohara H. Noda M. Nagasawa T. (2006). Maintenance of the hematopoietic stem cell pool by CXCL12-CXCR4 chemokine signaling in bone marrow stromal cell niches. *Immunity* 25 977–988. 10.1016/j.immuni.2006.10.016 17174120

[B205] Taichman R. S. Reilly M. J. Emerson S. G. (1996). Human osteoblasts support human hematopoietic progenitor cells in vitro bone marrow cultures. *Blood* 87 518–524. 10.1182/blood.v87.2.518.bloodjournal872518 8555473

[B206] Tefferi A. Vaidya R. Caramazza D. Finke C. Lasho T. Pardanani A. (2011). Circulating interleukin (IL)-8, IL-2R, IL-12, and IL-15 levels are independently prognostic in primary myelofibrosis: a comprehensive cytokine profiling study. *J. Clin. Oncol.* 29 1356–1363. 10.1200/jco.2010.32.9490 21300928

[B207] Teofili L. Martini M. Iachininoto M. G. Capodimonti S. Nuzzolo E. R. Torti L. (2011). Endothelial progenitor cells are clonal and exhibit the JAK2V617F mutation in a subset of thrombotic patients with Ph-negative myeloproliferative neoplasms. *Blood* 117 2700–2707. 10.1182/blood-2010-07-297598 21212285

[B208] Teofili L. Martini M. Nuzzolo E. R. Capodimonti S. Iachininoto M. G. Cocomazzi A. (2015). Endothelial progenitor cell dysfunction in myelodysplastic syndromes: possible contribution of a defective vascular niche to myelodysplasia. *Neoplasia* 17 401–409. 10.1016/j.neo.2015.04.001 26025663 PMC4468365

[B209] Thomas D. Majeti R. (2017). Biology and relevance of human acute myeloid leukemia stem cells. *Blood* 129 1577–1585. 10.1182/blood-2016-10-696054 28159741 PMC5364335

[B210] Thorén L. A. Liuba K. Bryder D. Nygren J. M. Jensen C. T. Qian H. (2008). Kit regulates maintenance of quiescent hematopoietic stem cells. *J. Immunol.* 180 2045–2053. 10.4049/jimmunol.180.4.2045 18250409

[B211] Tikhonova A. N. Dolgalev I. Hu H. Sivaraj K. K. Hoxha E. Cuesta-Domínguez Á, et al. (2019). The bone marrow microenvironment at single-cell resolution. *Nature* 569 222–228. 30971824 10.1038/s41586-019-1104-8 PMC6607432

[B212] Travaglino E. Benatti C. Malcovati L. Porta M. G. D. Gallì A. Bonetti E. (2008). Biological and clinical relevance of matrix metalloproteinases 2 and 9 in acute myeloid leukaemias and myelodysplastic syndromes. *Eur. J. Haematol.* 80 216–226. 10.1111/j.1600-0609.2007.01012.x 18081721

[B213] Trujillo A. McGee C. Cogle C. R. (2012). Angiogenesis in acute myeloid leukemia and opportunities for novel therapies. *J. Oncol.* 2012:128608. 10.1155/2012/128608 PMC3167188 21904549

[B214] Tzogani K. Penttilä K. Lapveteläinen T. Hemmings R. Koenig J. Freire J. (2020). EMA review of daunorubicin and cytarabine encapsulated in liposomes (Vyxeos, CPX−351) for the treatment of adults with newly diagnosed, therapy−related acute myeloid leukemia or acute myeloid leukemia with myelodysplasia−related changes. *Oncologist* 25 e1414–e1420. 32282100 10.1634/theoncologist.2019-0785 PMC7485353

[B215] Uckun F. M. Cogle C. R. Lin T. L. Qazi S. Trieu V. N. Schiller G. (2019). A phase 1B clinical study of combretastatin A1 diphosphate (OXi4503) and cytarabine (ARA-C) in combination (OXA) for patients with relapsed or refractory acute myeloid leukemia. *Cancers* 12:74. 10.3390/cancers12010074 31888052 PMC7016810

[B216] Upadhaya S. Krichevsky O. Akhmetzyanova I. Sawai C. M. Fooksman D. R. Reizis B. (2020). Intravital imaging reveals motility of adult hematopoietic stem cells in the bone marrow niche. *Cell Stem Cell* 27 336–345.e4. 32589864 10.1016/j.stem.2020.06.003 PMC7415613

[B217] Vanella L. Kim D. H. Asprinio D. Peterson S. J. Barbagallo I. Vanella A. (2010). HO-1 expression increases mesenchymal stem cell-derived osteoblasts but decreases adipocyte lineage. *Bone* 46 236–243. 10.1016/j.bone.2009.10.012 19853072 PMC2818489

[B218] Vas V. Senger K. Dörr K. Niebel A. Geiger H. (2012). Aging of the microenvironment influences clonality in hematopoiesis. *PLoS One* 7:e42080. 10.1371/journal.pone.0042080 22879906 PMC3412859

[B219] Vasudev N. S. Reynolds A. R. (2014). Anti-angiogenic therapy for cancer: current progress, unresolved questions and future directions. *Angiogenesis* 17 471–494. 10.1007/s10456-014-9420-y 24482243 PMC4061466

[B220] Vey N. Delaunay J. Martinelli G. Fiedler W. Raffoux E. Prebet T. (2016). Phase I clinical study of RG7356, an anti-CD44 humanized antibody, in patients with acute myeloid leukemia. *Oncotarget* 7 32532–32542. 10.18632/oncotarget.8687 27081038 PMC5078031

[B221] Vijay V. Miller R. Vue G. S. Pezeshkian M. B. Maywood M. Ast A. M. (2019). Interleukin-8 blockade prevents activated endothelial cell mediated proliferation and chemoresistance of acute myeloid leukemia. *Leuk. Res.* 84 106180. 10.1016/j.leukres.2019.106180 31299413 PMC6857733

[B222] Vinhas R. Mendes R. Fernandes A. R. Baptista P. V. (2017). Nanoparticles—emerging potential for managing leukemia and lymphoma. *Front. Bioeng. Biotechnol.* 5:79. 10.3389/fbioe.2017.00079 29326927 PMC5741836

[B223] Visnjic D. Kalajzic Z. Rowe D. W. Katavic V. Lorenzo J. Aguila H. L. (2004). Hematopoiesis is severely altered in mice with an induced osteoblast deficiency. *Blood* 103 3258–3264. 10.1182/blood-2003-11-4011 14726388

[B224] Wakabayashi M. Miwa H. Shikami M. Hiramatsu A. Ikai T. Tajima E. (2004). Autocrine pathway of angiopoietins–Tie2 system in AML cells: association with phosphatidyl-inositol 3 kinase. *Hematol. J.* 5 353–360. 10.1038/sj.thj.6200410 15297853

[B225] Walkley C. R. Olsen G. H. Dworkin S. Fabb S. A. Swann J. McArthur G. A. (2007). A microenvironment-induced myeloproliferative syndrome caused by retinoic acid receptor γ deficiency. *Cell* 129 1097–1110. 10.1016/j.cell.2007.05.014 17574023 PMC1974882

[B226] Wang E.S. Fetterly G. J. Pitzonka L. Brady W. E. Tan W. Greene J. (2014). Phase 1 study of the angiopoietin 1/2 neutralizing peptibody, trebananib, in acute myeloid leukemia. *J. Clin. Oncol.* 32(suppl. 15) 7082–7082. 10.1200/jco.2014.32.15_suppl.7082

[B227] Wang L. Zhang H. Rodriguez S. Cao L. Parish J. Mumaw C. (2014). Notch-dependent repression of mir-155 in the bone marrow niche regulates hematopoiesis in an NF-κB-dependent manner. *Cell Stem Cell* 15 51–65. 10.1016/j.stem.2014.04.021 24996169 PMC4398997

[B228] Watarai M. Miwa H. Shikami M. Sugamura K. Wakabayashi M. Satoh A. (2002). Expression of endothelial cell-associated molecules in AML cells. *Leukemia* 16 112–119. 10.1038/sj.leu.2402326 11840270

[B229] Weidenaar A. C. Elst A. T. Kampen K. R. Meeuwsen-de Boe T. Kamps W. A. Schuringa J. J. (2013). Impaired long-term expansion and self-renewal potential of pediatric acute myeloid leukemia–initiating cells By PTK787/ZK 222584. *Mol. Cancer Res.* 11 339–348. 10.1158/1541-7786.mcr-12-0113 23393162

[B230] Weidenaar A. C. Elst A. T. Koopmans-Klein G. Rosati S. den Dunnen W. F. Meeuwsen-de Boer T. (2011). High acute myeloid leukemia derived VEGFA levels are associated with a specific vascular morphology in the leukemic bone marrow. *Cell. Oncol. (Dordr.)* 34 289–296. 10.1007/s13402-011-0017-9 21468688 PMC3162634

[B231] Wilson A. Laurenti E. Oser G. van der Wath R. C. Blanco-Bose W. Jaworski M. (2008). Hematopoietic stem cells reversibly switch from dormancy to self-renewal during homeostasis and repair. *Cell* 135 1118–1129. 10.1016/j.cell.2008.10.048 19062086

[B232] Wimazal F. Krauth M.-T. Vales A. Böhm A. Agis H. Sonneck K. (2006). Immunohistochemical detection of vascular endothelial growth factor (VEGF) in the bone marrow in patients with myelodysplastic syndromes: correlation between VEGF expression and the FAB category. *Leuk. Lymphoma* 47 451–460. 10.1080/10428190500353083 16396768

[B233] Winkler I. G. Barbier V. Nowlan B. Jacobsen R. N. Forristal C. E. Patton J. T. (2012). Vascular niche E-selectin regulates hematopoietic stem cell dormancy, self renewal and chemoresistance. *Nat. Med.* 18 1651–1657. 10.1038/nm.2969 23086476

[B234] Winkler I. G. Sims N. A. Pettit A. R. Barbier V. Nowlan B. Helwani F. (2010). Bone marrow macrophages maintain hematopoietic stem cell (HSC) niches and their depletion mobilizes HSCs. *Blood* 116 4815–4828. 10.1182/blood-2009-11-253534 20713966

[B235] Wolock S. L. Krishnan I. Tenen D. E. Matkins V. Camacho V. Patel S. (2019). Mapping distinct bone marrow niche populations and their differentiation paths. *Cell Rep.* 28 302–311.e5. 31291568 10.1016/j.celrep.2019.06.031 PMC6684313

[B236] Xie Y. Yin T. Wiegraebe W. He X. C. Miller D. Stark D. (2009). Detection of functional haematopoietic stem cell niche using real-time imaging. *Nature* 457 97–101. 10.1038/nature07639 19052548

[B237] Xu C. Gao X. Wei Q. Nakahara F. Zimmerman S. E. Mar J. (2018). Stem cell factor is selectively secreted by arterial endothelial cells in bone marrow. *Nat. Commun.* 9:2449. 10.1038/s41467-018-04726-3 PMC6015052 29934585

[B238] Yamazaki S. Ema H. Karlsson G. Yamaguchi T. Miyoshi H. Shioda S. (2011). Nonmyelinating schwann cells maintain hematopoietic stem cell hibernation in the bone marrow niche. *Cell* 147 1146–1158. 10.1016/j.cell.2011.09.053 22118468

[B239] Yao L. Yokota T. Xia L. Kincade P. W. McEver R. P. (2005). Bone marrow dysfunction in mice lacking the cytokine receptor gp130 in endothelial cells. *Blood* 106 4093–4101. 10.1182/blood-2005-02-0671 16118327 PMC1895244

[B240] Yee K. W. H. O’Farrell A. M. Smolich B. D. Cherrington J. M. McMahon G. Wait C. L. (2002). SU5416 and SU5614 inhibit kinase activity of wild-type and mutant FLT3 receptor tyrosine kinase. *Blood* 100 2941–2949. 10.1182/blood-2002-02-0531 12351406

[B241] Yoshihara H. Arai F. Hosokawa K. Hagiwara T. Takubo K. Nakamura Y. (2007). Thrombopoietin/MPL signaling regulates hematopoietic stem cell quiescence and interaction with the osteoblastic niche. *Cell Stem Cell* 1 685–697. 10.1016/j.stem.2007.10.020 18371409

[B242] Youn M. Huang H. Chen C. Kam S. Wilkes M. C. Chae H.-D. (2019). MMP9 inhibition increases erythropoiesis in RPS14-deficient del(5q) MDS models through suppression of TGF-β pathways. *Blood Adv.* 3 2751–2763. 10.1182/bloodadvances.2019000537 31540902 PMC6759738

[B243] Zahiragic L. Schliemann C. Bieker R. Thoennissen N. H. Burow K. Kramer C. (2007). Bevacizumab reduces VEGF expression in patients with relapsed and refractory acute myeloid leukemia without clinical antileukemic activity. *Leukemia* 21 1310–1312. 10.1038/sj.leu.2404632 17330095

[B244] Zhang J. Niu C. Ye L. Huang H. He X. Tong W.-G. (2003). Identification of the haematopoietic stem cell niche and control of the niche size. *Nature* 425 836–841. 10.1038/nature02041 14574412

[B245] Zhang J. Ye J. Ma D. Liu N. Wu H. Yu S. (2013). Cross-talk between leukemic and endothelial cells promotes angiogenesis by VEGF activation of the Notch/Dll4 pathway. *Carcinogenesis* 34 667–677. 10.1093/carcin/bgs386 23239744

[B246] Zhang Y. Zhai W. Zhao M. Li D. Chai X. Cao X. (2015). Effects of iron overload on the bone marrow microenvironment in mice. *PLoS One* 10:e0120219. 10.1371/journal.pone.0120219 25774923 PMC4361683

[B247] Zhao M. Perry J. M. Marshall H. Venkatraman A. Qian P. He X. C. (2014). Megakaryocytes maintain homeostatic quiescence and promote post-injury regeneration of hematopoietic stem cells. *Nat. Med.* 20 1321–1326. 10.1038/nm.3706 25326798

[B248] Zhao Z.-G. Xu W. Yu H.-P. Fang B.-L. Wu S.-H. Li F. (2012). Functional characteristics of mesenchymal stem cells derived from bone marrow of patients with myelodysplastic syndromes. *Cancer Lett.* 317 136–143. 10.1016/j.canlet.2011.08.030 22240014

[B249] Zheng Q. Zhao Y. Guo J. Zhao S. Fei C. Xiao C. (2018). Iron overload promotes mitochondrial fragmentation in mesenchymal stromal cells from myelodysplastic syndrome patients through activation of the AMPK/MFF/Drp1 pathway. *Cell Death Dis.* 9:515. 10.1038/s41419-018-0552-7 PMC5938711 29725013

[B250] Zhou B. O. Ding L. Morrison S. J. (2015). Hematopoietic stem and progenitor cells regulate the regeneration of their niche by secreting Angiopoietin-1. *Elife* 4:e05521. 10.7554/eLife.05521 PMC4411515 25821987

[B251] Zhou B. O. Yu H. Yue R. Zhao Z. Rios J. J. Naveiras O. (2017). Bone marrow adipocytes promote the regeneration of stem cells and haematopoiesis by secreting SCF. *Nat. Cell Biol.* 19 891–903. 10.1038/ncb3570 28714970 PMC5536858

[B252] Zhou B. O. Yue R. Murphy M. M. Peyer J. G. Morrison S. J. (2014). Leptin-receptor-expressing mesenchymal stromal cells represent the main source of bone formed by adult bone marrow. *Cell Stem Cell* 15 154–168. 10.1016/j.stem.2014.06.008 24953181 PMC4127103

[B253] Zhu J. Garrett R. Jung Y. Zhang Y. Kim N. Wang J. (2007). Osteoblasts support B-lymphocyte commitment and differentiation from hematopoietic stem cells. *Blood* 109 3706–3712. 10.1182/blood-2006-08-041384 17227831

[B254] Zhu R.-J. Wu M.-Q. Li Z.-J. Zhang Y. Liu K.-Y. (2013). Hematopoietic recovery following chemotherapy is improved by BADGE-induced inhibition of adipogenesis. *Int. J. Hematol.* 97 58–72. 10.1007/s12185-012-1233-4 23264188

[B255] Zon L. I. (2008). Intrinsic and extrinsic control of haematopoietic stem-cell self-renewal. *Nature* 453 306–313. 10.1038/nature07038 18480811

