# Supplementary material for: The Dynamic Interface Between the Bone Marrow Vascular Niche and Hematopoietic Stem Cells in Myeloid Malignancy
Source: Front Cell Dev Biol. 2021 Mar 11;9:635189. doi: 10.3389/fcell.2021.635189 (PMC7991089; doi:10.3389/fcell.2021.635189)
Supplement: Supplementary file 1 [file Table_1.docx]

**Table 1 Cre-inducible murine models used to study the vascular niche**

| Cre line | Cell specificity | Inducible | References |
| --- | --- | --- | --- |
| Tie2-Cre | Pan-ECs (and hematopoietic cells) | No | [1] |
| Flk1‐Cre | Pan-ECs (and hematopoietic cells) | No | [2] |
| Cdh5‐Cre | Pan-ECs (and hematopoietic cells) | No | [3] |
| Tie2‐CreERT2 | Pan-ECs | Yes (tamoxifen) | [4] |
| Cdh5-CreERT2 | Pan-ECs | Yes (tamoxifen) | [5] |
| Cdh5(PAC)‐CreERT2 | Pan-ECs | Yes (tamoxifen) | [6] |
| Endothelial‐SCL‐CreERT | Pan-ECs | Yes (tamoxifen) | [7] |
| Bmx-CreERT2 | Arterial ECs | Yes (tamoxifen) | [8, 9] |
| EpoR-Cre | Sinusoidal ECs (and erythroid cells) | No | [8, 10] |
| LepR-Cre | Nestin^low^/Cxcl12^hi^ perivascular MSCs | No | [11, 12] |
| Ng2-Cre | Nestin^hi^/Cxcl12^lo^ perivascular MSCs | No | [11, 13] |
| Nes-Cre | Perivascular MSCs (and nervous system/ECs depending on the strain) | No | [14, 15] |
| Nestin-creERT2 | Perivascular MSCs | Yes (tamoxifen) | [16, 17] |
| Prx1-Cre | Perivascular MSCs | No | [18] |
| Prx1-CreER-GFP | Perivascular MSCs | Yes (tamoxifen) | [19] |
| Osx1-GFP::Cre | Osteoprogenitors | No but doxycycline-repressible | [20] |
| Osx-CreERT2 | Osteoprogenitors | Yes (tamoxifen) | [21, 22] |
| Col2.3-Cre | Mature osteoblasts | No | [23] |
| Mx1-Cre | Hematopoietic cells (and some perivascular MSCs) | Yes (poly I:C) | [24] |
| Vav-Cre | Hematopoietic cells (and ECs) | No | [25-27] |
| Vav-iCre | Hematopoietic cells | No | [25-30] |
| HSC-SCL-Cre-ERT | HSPCs | Yes (tamoxifen) | [31] |

*ECs, endothelial cells; MSCs, mesenchymal stem cells; HSPCs, hematopoietic stem and progenitor cells; poly I:C, polyinosinic:polycytidylic acid*

**Supplementary References**

1. Kisanuki, Y.Y., R.E. Hammer, J. Miyazaki , S.C. Williams, J.A. Richardson, and M. Yanagisawa. Tie2-Cre transgenic mice: a new model for endothelial cell-lineage analysis in vivo*.* *Dev Biol* (2001); 230(2): 230-42.

2. Licht, A.H., S. Raab, U. Hofmann, and G. Breier. Endothelium-specific Cre recombinase activity in flk-1-Cre transgenic mice*.* *Dev Dyn* (2004); 229(2): 312-8.

3. Alva, J.A., A.C. Zovein, A. Monvoisin, T. Murphy, A. Salazar, N.L. Harvey, et al. VE-Cadherin-Cre-recombinase transgenic mouse: a tool for lineage analysis and gene deletion in endothelial cells*.* *Dev Dyn* (2006); 235(3): 759-67.

4. Forde, A., R. Constien, H.J. Gröne, G. Hämmerling, and B. Arnold. Temporal Cre-mediated recombination exclusively in endothelial cells using Tie2 regulatory elements*.* *Genesis* (2002); 33(4): 191-7.

5. Monvoisin, A., J.A. Alva, J.J. Hofmann, A.C. Zovein, T.F. Lane, and M.L. Iruela-Arispe. VE-cadherin-CreERT2 transgenic mouse: a model for inducible recombination in the endothelium*.* *Dev Dyn* (2006); 235(12): 3413-22.

6. Wang, Y., M. Nakayama, M.E. Pitulescu, T.S. Schmidt, M.L. Bochenek, A. Sakakibara, et al. Ephrin-B2 controls VEGF-induced angiogenesis and lymphangiogenesis*.* *Nature* (2010); 465(7297): 483-6.

7. Göthert, J.R., S.E. Gustin, J.A. van Eekelen, U. Schmidt, M.A. Hall, S.M. Jane, et al. Genetically tagging endothelial cells in vivo: bone marrow-derived cells do not contribute to tumor endothelium*.* *Blood* (2004); 104(6): 1769-77.

8. Xu, C., X. Gao, Q. Wei, F. Nakahara, S.E. Zimmerman, J. Mar, et al. Stem cell factor is selectively secreted by arterial endothelial cells in bone marrow*.* *Nat Commun* (2018); 9(1): 2449.

9. Ehling, M., S. Adams, R. Benedito, and R.H. Adams. Notch controls retinal blood vessel maturation and quiescence*.* *Development* (2013); 140(14): 3051-61.

10. Heinrich, A.C., R. Pelanda, and U. Klingmüller. A mouse model for visualization and conditional mutations in the erythroid lineage*.* *Blood* (2004); 104(3): 659-66.

11. Asada, N., Y. Kunisaki, H. Pierce, Z. Wang, N.F. Fernandez, A. Birbrair, et al. Differential cytokine contributions of perivascular haematopoietic stem cell niches*.* *Nat Cell Biol* (2017); 19(3): 214-223.

12. DeFalco, J., M. Tomishima, H. Liu, C. Zhao, X. Cai, J.D. Marth, et al. Virus-assisted mapping of neural inputs to a feeding center in the hypothalamus*.* *Science* (2001); 291(5513): 2608-13.

13. Zhu, X., D.E. Bergles, and A. Nishiyama. NG2 cells generate both oligodendrocytes and gray matter astrocytes*.* *Development* (2008); 135(1): 145-57.

14. Tronche, F., C. Kellendonk, O. Kretz, P. Gass, K. Anlag, P.C. Orban, et al. Disruption of the glucocorticoid receptor gene in the nervous system results in reduced anxiety*.* *Nat Genet* (1999); 23(1): 99-103.

15. Trumpp, A., M.J. Depew, J.L. Rubenstein, J.M. Bishop, and G.R. Martin. Cre-mediated gene inactivation demonstrates that FGF8 is required for cell survival and patterning of the first branchial arch*.* *Genes Dev* (1999); 13(23): 3136-48.

16. Battiste, J., A.W. Helms, E.J. Kim, T.K. Savage, D.C. Lagace, C.D. Mandyam, et al. Ascl1 defines sequentially generated lineage-restricted neuronal and oligodendrocyte precursor cells in the spinal cord*.* *Development* (2007); 134(2): 285-93.

17. Lagace, D.C., M.C. Whitman, M.A. Noonan, J.L. Ables, N.A. DeCarolis, A.A. Arguello, et al. Dynamic contribution of nestin-expressing stem cells to adult neurogenesis*.* *J Neurosci* (2007); 27(46): 12623-9.

18. Logan, M., J.F. Martin, A. Nagy, C. Lobe, E.N. Olson, and C.J. Tabin. Expression of Cre Recombinase in the developing mouse limb bud driven by a Prxl enhancer*.* *Genesis* (2002); 33(2): 77-80.

19. Kawanami, A., T. Matsushita, Y.Y. Chan, and S. Murakami. Mice expressing GFP and CreER in osteochondro progenitor cells in the periosteum*.* *Biochem Biophys Res Commun* (2009); 386(3): 477-82.

20. Rodda, S.J. and A.P. McMahon. Distinct roles for Hedgehog and canonical Wnt signaling in specification, differentiation and maintenance of osteoblast progenitors*.* *Development* (2006); 133(16): 3231-44.

21. Maes, C., T. Kobayashi, and H.M. Kronenberg. A novel transgenic mouse model to study the osteoblast lineage in vivo*.* *Ann N Y Acad Sci* (2007); 1116: 149-64.

22. Maes, C., T. Kobayashi, M.K. Selig, S. Torrekens, S.I. Roth, S. Mackem, et al. Osteoblast precursors, but not mature osteoblasts, move into developing and fractured bones along with invading blood vessels*.* *Dev Cell* (2010); 19(2): 329-44.

23. Dacquin, R., M. Starbuck, T. Schinke, and G. Karsenty. Mouse alpha1(I)-collagen promoter is the best known promoter to drive efficient Cre recombinase expression in osteoblast*.* *Dev Dyn* (2002); 224(2): 245-51.

24. Kühn, R., F. Schwenk, M. Aguet, and K. Rajewsky. Inducible gene targeting in mice*.* *Science* (1995); 269(5229): 1427-9.

25. Croker, B.A., D. Metcalf, L. Robb, W. Wei, S. Mifsud, L. DiRago, et al. SOCS3 is a critical physiological negative regulator of G-CSF signaling and emergency granulopoiesis*.* *Immunity* (2004); 20(2): 153-65.

26. de Boer, J., A. Williams, G. Skavdis, N. Harker, M. Coles, M. Tolaini, et al. Transgenic mice with hematopoietic and lymphoid specific expression of Cre*.* *Eur J Immunol* (2003); 33(2): 314-25.

27. Georgiades, P., S. Ogilvy, H. Duval, D.R. Licence, D.S. Charnock-Jones, S.K. Smith, et al. VavCre transgenic mice: a tool for mutagenesis in hematopoietic and endothelial lineages*.* *Genesis* (2002); 34(4): 251-6.

28. Ogilvy, S., D. Metcalf, L. Gibson, M.L. Bath, A.W. Harris, and J.M. Adams. Promoter elements of vav drive transgene expression in vivo throughout the hematopoietic compartment*.* *Blood* (1999); 94(6): 1855-63.

29. Shimshek, D.R., J. Kim, M.R. Hübner, D.J. Spergel, F. Buchholz, E. Casanova, et al. Codon-improved Cre recombinase (iCre) expression in the mouse*.* *Genesis* (2002); 32(1): 19-26.

30. Siegemund, S., J. Shepherd, C. Xiao, and K. Sauer. hCD2-iCre and Vav-iCre mediated gene recombination patterns in murine hematopoietic cells*.* *PLoS One* (2015); 10(4): e0124661.

31. Göthert, J.R., S.E. Gustin, M.A. Hall, A.R. Green, B. Göttgens, D.J. Izon, et al. In vivo fate-tracing studies using the Scl stem cell enhancer: embryonic hematopoietic stem cells significantly contribute to adult hematopoiesis*.* *Blood* (2005); 105(7): 2724-32.
